# Ruthenium-based PACT agents based on bisquinoline chelates: synthesis, photochemistry, and cytotoxicity

**DOI:** 10.1007/s00775-021-01882-8

**Published:** 2021-08-10

**Authors:** Anja Busemann, Ingrid Flaspohler, Xue-Quan Zhou, Claudia Schmidt, Sina K. Goetzfried, Vincent H. S. van Rixel, Ingo Ott, Maxime A. Siegler, Sylvestre Bonnet

**Affiliations:** 1 Leiden Institute of Chemistry, Leiden University, Einsteinweg 55, 2333CC Leiden, The Netherlands; 2 Institute of Medicinal and Pharmaceutical Chemistry, Technische Universität Braunschweig, Beethovenstrasse 55, 38106 Braunschweig, Germany; 3 Small Molecule X-Ray Facility, Department of Chemistry, Johns Hopkins University, Baltimore, Maryland 21218 USA

## Abstract

The known ruthenium complex [Ru(tpy)(bpy)(Hmte)](PF_6_)_2_ ([**1**](PF_6_)_2_, where tpy = 2,2’:6’,2″-terpyridine, bpy = 2,2’-bipyridine, Hmte = 2-(methylthio)ethanol) is photosubstitutionally active but non-toxic to cancer cells even upon light irradiation. In this work, the two analogs complexes [Ru(tpy)(NN)(Hmte)](PF_6_)_2_, where NN = 3,3'-biisoquinoline (i-biq, [**2**](PF_6_)_2_) and di(isoquinolin-3-yl)amine (i-Hdiqa, [**3**](PF_6_)_2_), were synthesized and their photochemistry and phototoxicity evaluated to assess their suitability as photoactivated chemotherapy (PACT) agents. The increase of the aromatic surface of [**2**](PF_6_)_2_ and [**3**](PF_6_)_2_, compared to [**1**](PF_6_)_2_, leads to higher lipophilicity and higher cellular uptake for the former complexes. Such improved uptake is directly correlated to the cytotoxicity of these compounds in the dark: while [**2**](PF_6_)_2_ and [**3**](PF_6_)_2_ showed low EC_50_ values in human cancer cells, [**1**](PF_6_)_2_ is not cytotoxic due to poor cellular uptake. While stable in the dark, all complexes substituted the protecting thioether ligand upon light irradiation (520 nm), with the highest photosubstitution quantum yield found for [**3**](PF_6_)_2_ (Φ_[**3**]_ = 0.070). Compounds [**2**](PF_6_)_2_ and [**3**](PF_6_)_2_ were found both more cytotoxic after light activation than in the dark, with a photo index of 4. Considering the very low singlet oxygen quantum yields of these compounds, and the lack of cytotoxicity of the photoreleased Hmte thioether ligand, it can be concluded that the toxicity observed after light activation is due to the photoreleased aqua complexes [Ru(tpy)(NN)(OH_2_)]^2+^, and thus that [**2**](PF_6_)_2_ and [**3**](PF_6_)_2_ are promising PACT candidates.

**Graphic abstract:**

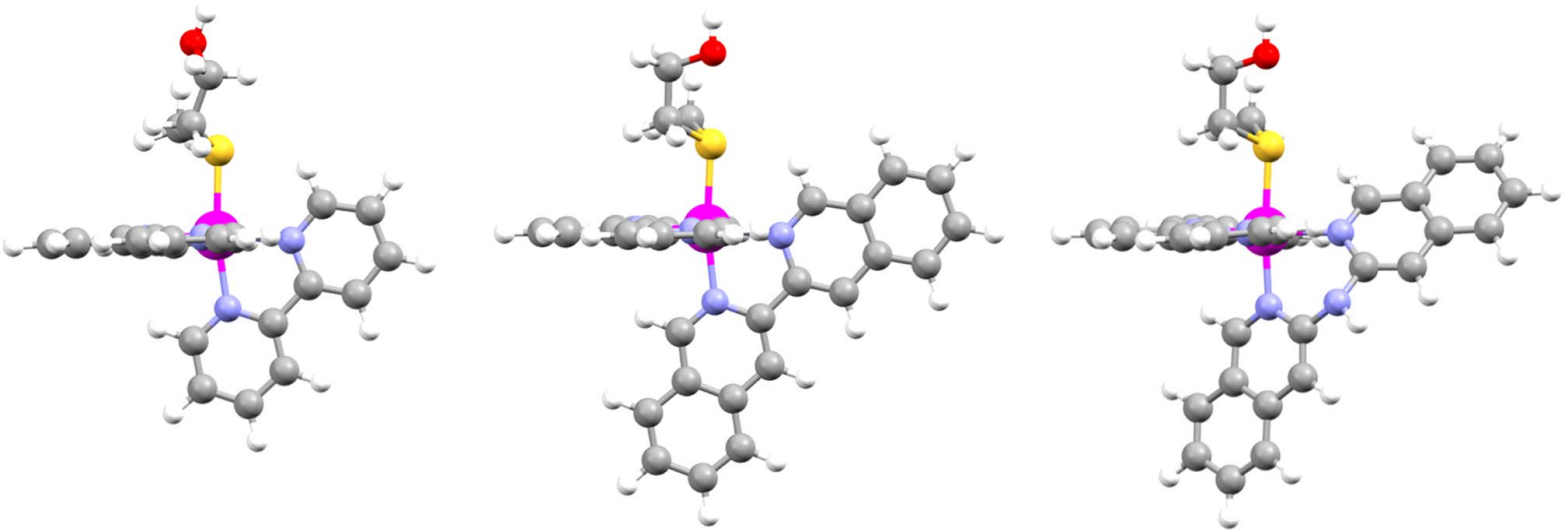

**Supplementary Information:**

The online version contains supplementary material available at 10.1007/s00775-021-01882-8.

## Introduction

In recent years, ruthenium polypyridyl complexes gained attention in the field of phototherapy for their favorable photophysical and photochemical properties [1]. Drug activation by light irradiation at the tumor site provides physical selectivity towards cancerous tissues and minimizes the effect of the drug on the healthy, non-irradiated tissues. Therefore, undesired side effects are expected to be reduced. Two different types of phototherapy are distinguished: photodynamic therapy (PDT) and photoactivated chemotherapy (PACT). In both cases, a molecule is promoted to a singlet metal-to-ligand charge transfer excited state (^1^MLCT) by photon absorption. From there, the molecule undergoes intersystem crossing (ISC) to a triplet metal-to-ligand charge transfer excited state (^3^MLCT). This ^3^MLCT state can be deactivated via four different pathways: non-radiative deactivation, emission of a photon, energy transfer to molecular oxygen to generate singlet oxygen (^1^O_2_), or thermal population of a low-lying triplet metal-centered excited state (^3^MC), which leads to ligand photosubstitution [1–7]. In PDT, the production of ^1^O_2_ leads to serious oxidative damage of the cells, culminating in cell death. In PACT, on the other hand, the prodrug, which is usually poorly toxic in the dark, is activated by ligand photosubstitution [6, 8–12]. The activated drug becomes capable of interacting with biomolecules, causing cell death in an oxygen-independent way [7, 10, 13–16]. Since thermal promotion from the photochemically generated ^3^MLCT state into the photosubstitutionally active ^3^MC state is a competitive pathway for the quenching of the ^3^MLCT state, good PACT agents are usually not emissive and produce only small amounts of ^1^O_2_ [17].

To be a promising PACT agent, a metal complex has to fulfill three criteria: (1) it should be thermally stable in solution in the dark, (2) it should be photoactivatable with acceptable photosubstitution quantum yields, typically in the order of **Φ** ~ 0.01–0.05, and (3) it should show an increased cytotoxicity after light activation, compared to the dark. For example, [Ru(tpy)(bpy)(Hmte)](PF_6_)_2_ ([**1**](PF_6_)_2_, where tpy = 2,2’:6’,2″-terpyridine, bpy = 2,2’-bipyridine, and Hmte = 2-(methylthio)ethanol), is known to undergo photosubstitution of the thioether Hmte ligand under blue light irradiation, to generate an aqua ruthenium-based photoproduct [Ru(tpy)(bpy)(OH_2_)]^2+^ [18] that is known to be non-cytotoxic [19]. It is hence a good example for a *chemically* activated compound, i.e., a compound capable of photosubstitution, that is not expected to be *biologically* activated because its photoactivated product is not cytotoxic. To obtain high phototoxicity after light activation, ruthenium complexes require efficient cellular uptake, as well as some form of deleterious interaction of the activated photoproducts with biological molecules. Bi-cationic polypyridyl ruthenium complexes such as [**1**](PF_6_)_2_ often show low cellular uptake [20], can be solved either by lowering the positive charge of the complex, e.g. via cyclometallation [21, 22], or by increasing the hydrophobicity of the ligands, e.g. by expanding the aromatic surface of its polypyridyl ligands or adding methyl groups [23, 24]. On the other hand, too lipophilic complexes may show too high dark cytotoxicity, which is a problem in phototherapy [25]. For PACT compounds, ligand expansion aimed at increasing steric hindrance and thus photosubstitution efficacy [26, 27], may also lead to distorted complex geometries, resulting in uncontrolled ligand release, thus thermal activation in the dark [18, 24, 28]. Overall, the design of a good PACT compound requires careful balancing of the lipophilicity of the complex and its photoreactivity.

**Fig. 1:**
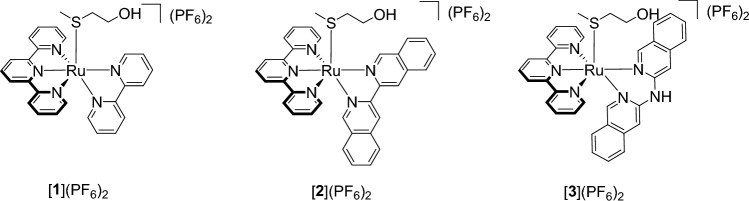
Chemical structures of the ruthenium-based PACT agents [**1**](PF_6_)_2_–[**3**](PF_6_)_2_

In this work, two new ruthenium-based PACT compounds with the formula [Ru(tpy)(NN)(Hmte)](PF_6_)_2_ (where NN = i-biq (3,3'-biisoquinoline), [**2**](PF_6_)_2_; or i-Hdiqa (di(isoquinolin-3-yl)amine), [**3**](PF_6_)_2_); Fig. 1), are reported. The increased aromatic surface of the bidentate ligands, compared to bpy, was chosen to improve cellular uptake. In addition, the dipyridylamine (Hdpa) scaffold, on which i-Hdiqa is based, has been shown to play a role in cellular uptake, compared to bpy-based systems [29]. Considering the promising results obtained with the tetrapyridyl complex [Ru(H_2_biqbpy)(dmso)(Cl)]^+^, where H_2_biqbpy = 6,6′-bis[*N*-(isoquinolyl)-1-amino]-2,2′-bipyridine [30], an amine bridge was introduced here to the i-biq ligand resulting in the i-Hdiqa analog, thereby extending the family of [Ru(tpy)(NN)(SRR’)]^2+^ complexes studied for PACT [17]. Next to cellular uptake, the enlarged aromatic rings of the ligands i-biq and i-Hdiqa may also enhance interaction of the complex with proteins, membranes, or DNA, which may lead to improved cytotoxicity [31]. The monodentate thioether ligand Hmte, on the other hand, provides excellent thermal stability in the dark, while offering good photochemical release [18]. The synthesis, photochemistry, cytotoxicity, and cellular uptake of these compounds are reported, and compared to that of the known complex [**1**](PF_6_)_2_.

## Results and discussion

### Synthesis and characterization

The bidentate ligand i-biq was obtained following a reported procedure [32]. The ligand i‑Hdiqa is also known [33] and was synthesized using a Buchwald-Hartwig coupling as described for the synthesis of other dipyridylamine derivatives in literature [34]. After purification by column chromatography, the ligand was isolated as NMR-pure solid in 48% yield. The two ruthenium-based PACT compounds [**2**](PF_6_)_2_ and [**3**](PF_6_)_2_ were synthesized following the same reaction route as for [**1**](PF_6_)_2_ (Scheme S1). In short, the bidentate ligand was first coordinated to the ruthenium precursor [Ru(tpy)(Cl)_3_], before the monodentate chloride ligand was thermally substituted by the protecting thioether ligand Hmte. The desired complexes were obtained in good yield (50 and 60%, respectively), and their purity was confirmed with ^1^H NMR, ^13^C NMR, and elemental analysis. [**1**](PF_6_)_2_ was found much more soluble in water (log P_ow_ = − 3.28 ± 0.31), compared to [**3**](PF_6_)_2_ which had intermediate hydrophilicity (log P_ow_ = 0.45 ± 0.10), and [**2**](PF_6_)_2_ which was the most hydrophobic complex of the series (log P_ow_ = 2.10 ± 0.27, see Table S1). These values demonstrate not only the expectedly increased lipophilicity of the i-biq and i-Hdiqa ligands, compared to bpy, but also the significant polarity, compared to [**2**](PF_6_)_2_, generated in [**3**](PF_6_)_2_ by the non-coordinated amine bridge.

**Fig. 2:**
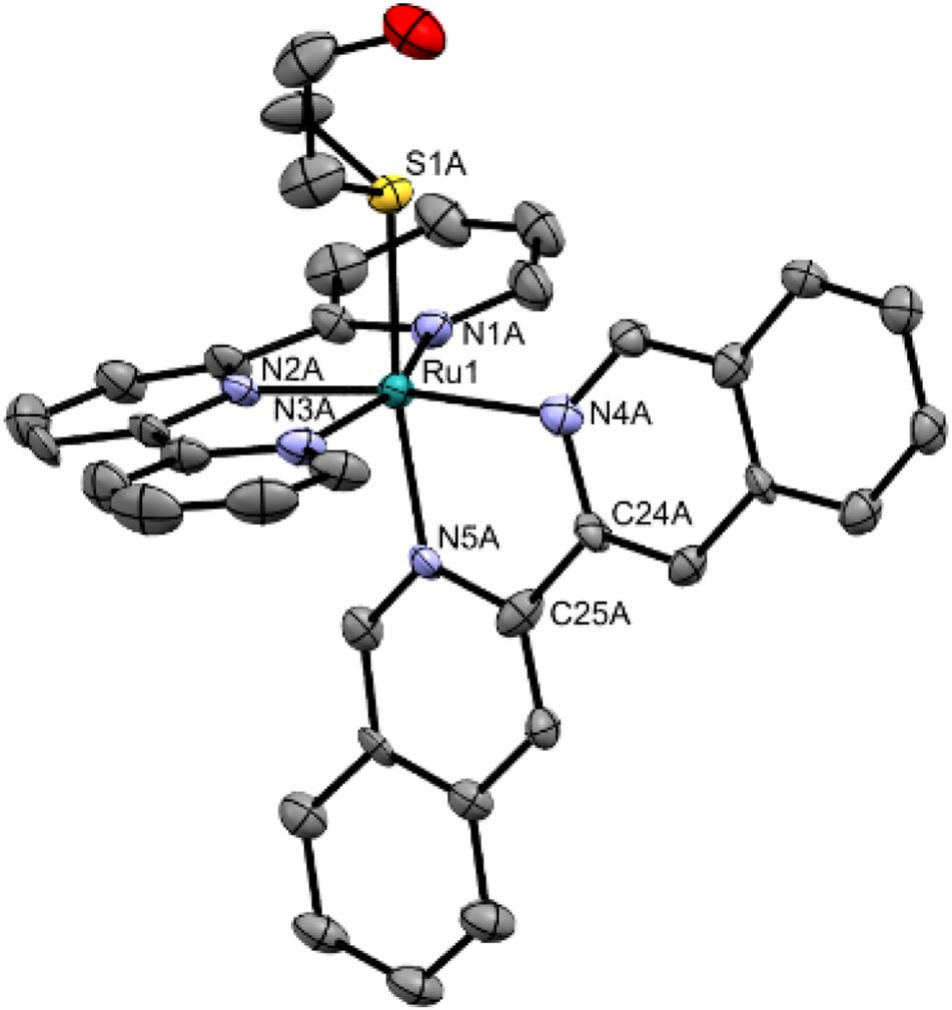
Displacement ellipsoid (50% probability level) of one crystallographically independent cationic part as observed in the crystal structure of [**2**]^2+^. The other cation, disorder, counter ions, and H atoms have been omitted for clarity

Single crystals suitable for X-ray structure determination of complex [**2**](PF_6_)_2_ were obtained in the dark by slow vapor diffusion of diisopropyl ether in an acetonitrile solution of the complex (Fig. 2). Selected bond lengths, angles, and torsion angles are summarized in Table 1 and are compared to those of [**1**](PF_6_)_2_ [18]. The coordination bond lengths of the i-biq complex are not significantly different from those with bpy *e.g.* Ru-N4 is 2.104(10) *vs.* 2.092(1) Å for [**2**](PF_6_)_2_ vs. [**1**](PF_6_)_2_. The torsion angle of the coordinated i-biq is slightly smaller than that of bpy (N4-C24-C25-N5 = 1.9(14)° vs. N4-C20-C21-N5 = 5.3(2)°, Table 1). The Hmte ligand is bound via the sulfur atom to ruthenium, with similar bond lengths for both complexes (Ru-S = 2.368(3) and 2.3690(5) for [**2**](PF_6_)_2_ and [**1**](PF_6_)_2_, respectively). As single crystals for complex [**1**](PF_6_)_2_ could not be obtained, density functional theory (DFT) was used to compare the structure of [**1**]^2+^, [**2**]^2+^, and [**3**]^2+^ (Fig. 2; Table S4-S6). The bond distances and angles of the DFT models of [**2**]^2+^ and [**3**]^2+^ are also provided in Table 1. For [**2**]^2+^, the minimized geometry of the DFT model was very close to that of the X-ray structure. For [**3**]^2+^, no significant differences in bond lengths or angles are found compared to [**2**]^2+^, however, the position of the bidentate ligand towards the tpy ligand does differ. While i-biq is perpendicular to the tpy ligand, i-Hdiqa shows a characteristic bending at the amine bridge (Figure S11) [35, 36]. Calculations of the bond angle variance (*σ*^2^ = 60.3 and 46.4, respectively) [37], and the mean quadratic elongation (*λ* = 3.65 and 3.46, respectively) [38], revealed that the octahedral geometry of both complexes is distorted, but that this distortion is mostly caused by the coordination of the tpy ligand (N1-Ru1-N3 = 158.17 and 158.01°, respectively). Overall, the extension of the bpy ligand into i-biq or i-Hdiqa does not lead to significant changes of the coordination sphere or bond lengths to the ruthenium ion.

**Table 1:** Selected bond lengths (Å), angles (°), and torsion angles (°) for [**1**](PF_6_)_2_–[**3**](PF_6_)_2_

	[**1**](PF_6_)_2_^a^	[**2**](PF_6_)_2_^b^	[**1**]^2+c^	[**2**]^2+c^	[**3**]^2+c^
Ru-S1	2.3690(5)	2.368(3)	2.394	2.396	2.396
Ru-N1	2.061(1)	2.071(9)	2.093	2.094	2.095
Ru-N2	1.961(1)	1.967(10)	1.979	1.979	1.978
Ru-N3	2.066(1)	2.073(10)	2.091	2.096	2.114
Ru-N4	2.092(1)	2.104(10)	2.116	2.117	2.138
Ru-N5	2.064(1)	2.074(9)	2.080	2.082	2.115
N1-Ru1-N2	80.08(6)	79.3(4)	79.10	79.14	79.17
N2-Ru1-N3	79.39(6)	80.1(4)	79.11	79.19	78.90
N1-Ru1-N3	159.31(6)	159.4(4)	158.06	158.17	158.01
N4-Ru1-N5	78.12(6)	79.4(4)	77.65	78.43	86.45
N4-C20-C21-N5	5.3(2)	—	—	—	—
N4-C24-C25-N5	—	1.9(14)	0.22	4.46	—
*λ*^d^	3.61	3.42	3.61	3.65	3.46
*σ*^2e^	57.3	58.6	62.2	60.3	46.4

^a^Data from Bahreman et al. [18];

^b^Data obtained by X-ray analysis (provided only for the crystallographically independent cation labeled A in the asymmetric unit of [**2**](PF_6_)_2_)

^c^Data from DFT calculations at the PBE0/TZP/COSMO level in water

^d^Mean quadratic elongation, where *d*_n_ is one of the six bond lengths and < *d* > is the mean of those bond lengths

^e^Bond angle variance where *θ*_n_ is one of the twelve angles

### Photochemistry

The two complexes have many overlapping ^1^MLCT absorption bands extending between 400 and up to 600 nm, with an absorption maximum at 429 nm for [2]^2+^ and a shoulder near 470 nm for [**3**]^2+^, while for [**1**](PF_6_)_2_ the maximum appeared at 450 nm (Table 2; Figure S2). The low-energy transitions for [**1**]^2+^ and [**2**]^2+^ were very similar, confirming the electronic similarity between bpy and i-biq, while i-Hdiqa-based [**3**]^2+^ showed overall bathochromically shifted absorption bands extending in the red region of the spectrum. The hypothesis that such a shift may be caused by the bending of the i-Hdiqa ligand, was confirmed by TDDFT calculations at the PBE0/TZP/COSMO(water) level of theory. The first significant transition (*f* > 0.01) for [**1**]^2+^ and [**2**]^2+^, calculated at 451 and 461 nm, respectively (Table S3), transferred an electron from an essentially metal-centered 3d_xz_ to the π* orbital centered on terpyridine (Figure S12a–b). In contrast, for [**3**]^2+^ the metal-based 3d orbital was much more in the xy plane of the terpyridine ligand, and significantly mixed via antibonding orbital overlap with the π system of the bent quinoline moiety of the i-Hdiqa ligand (Figure S12c), thereby reducing the energy of the ^1^MLCT transition into the terpyridine π*-based orbital, which hence appeared at a bathochromically shifted wavelength (476 nm). Overall, in [**2**]^2+^ the extension of the conjugation of the bpy system, compared to [**1**]^2+^, does not significantly influence the lowest-energy ^1^MLCT transition of the complex as this transition involves the terpyridine ligand and not the bidentate chelate, while in [**3**]^2+^ the formation of a 6-membered metallacycle due to the presence of the additional NH bridge, generates a distortion of the planarity of the i-Hdiqa ligand that destabilizes the HOMO, thereby shifting the lowest-energy ^1^MLCT transitions towards the red region of the spectrum.

**Table 2:** Lowest-energy absorption maxima (*λ*_max_ in nm), molar absorption coefficients at *λ*_max_ (*ε*_max_ in M^−1^ · cm^−1^) in water, singlet oxygen generation quantum yields (Φ_Δ_) in aerated methanol-*d*_4_, phosphorescence quantum yields (Φ_P_) in aerated methanol-*d*_4_, and photosubstitution quantum yields upon irradiation at 517 nm (Φ_517_) in water for complexes [**1**](PF_6_)_2_–[**3**](PF_6_)_2_

Complex	NN	*λ*_max_ (*ε*_max_)^a^	Φ_P_^b^	Φ_Δ_^b^	Φ_517_^a^
[**1**](PF_6_)_2_	bpy	450 (6.60 · 10^3^)^c^	< 1.0 · 10^−4d^	< 0.005^d^	0.022^c^
[**2**](PF_6_)_2_	i-biq	429 (5.76 · 10^3^)	1.5 · 10^−4^	0.010	0.023
[**3**](PF_6_)_2_	i-Hdiqa	470 (5.35 · 10^3^)	4.5 · 10^−4^	0.042	0.077

^a^In water

^b^In methanol-*d*_4_;[39]

^c^Data taken from Bahreman et al. [18]

^d^Data Busemann et al. [40]

**Fig. 3:**
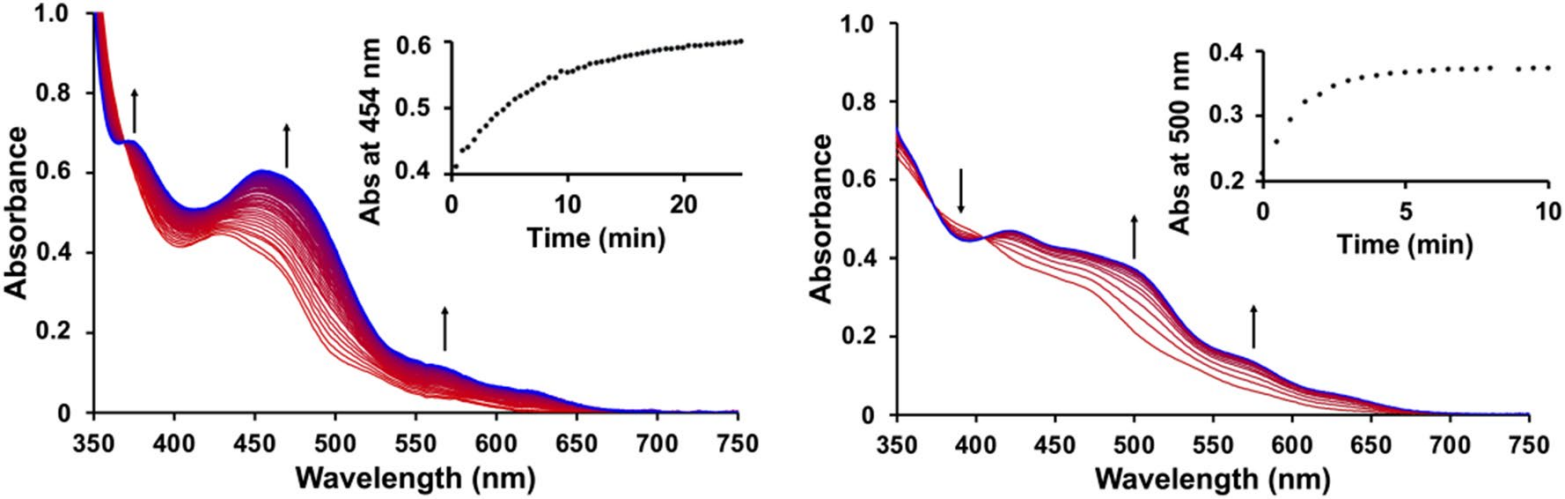
Evolution of the UV–vis absorption spectra of a solution of [**2**](PF_6_)_2_ (left) and [**3**](PF_6_)_2_ (right) upon green light irradiation in water. Conditions: [Ru] = 0.074 and 0.061 mM for [**2**](PF_6_)_2_ and [**3**](PF_6_)_2_, respectively, *T* = 37 °C, light source: λ = 517 nm, Δλ_1/2_ = 23 nm, 5.2 mW, photon flux Φ = 5.2 · 10^−8^ mol · s^−1^ for [**2**](PF_6_)_2_ and [**3**](PF_6_)_2_, *V* = 3 mL, under air atmosphere. Inset: time evolution of absorbance at wavelength 454 nm for [**2**](PF_6_)_2_ and 500 nm for [**3**](PF_6_)_2_

**Scheme 1:**
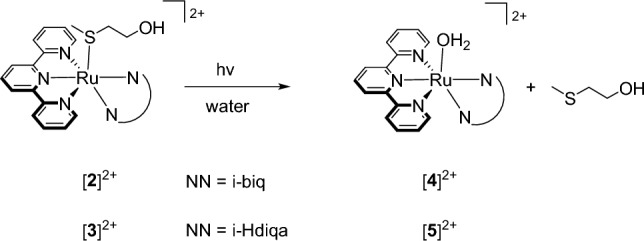
Photosubstitution of the protecting Hmte ligand in [Ru(tpy)(NN)(Hmte)]^2+^ ([**2**]^2+^ and [**3**]^2+^) to form the corresponding aqua species [Ru(tpy)(NN)(OH_2_)]^2+^ ([**4**]^2+^ and [**5**]^2+^)

Although [**2**](PF_6_)_2_ and [**3**](PF_6_)_2_ were perfectly stable in pure water in the dark at 37 °C for 24 h (Figure S1a and S1b), they were clearly chemically photoactivated. Their photoreactivity was investigated upon green light irradiation (517 nm) in water at 37 °C using UV–vis spectroscopy (Fig. 3). For each complex, upon irradiation a typical bathochromic shift of the absorption maximum was observed, due to the release of the thioether ligand and the formation of the corresponding aqua complex [Ru(tpy)(NN)(OH_2_)]^2+^ ([**4**]^2+^ and [**5**]^2+^ for NN = i-biq and i-Hdiqa, respectively, see Scheme 1) [17, 41, 42]. The formation of the aqua complexes was confirmed with mass spectrometry (Figure S4). The UV–vis spectra recorded during irradiation showed isosbestic points (at 369; 375 and 404, respectively), indicating a one-step photosubstitution reaction. The Glotaran software package was used to fit the time evolution of the UV–vis absorption spectra to a single photoreaction, and to obtain the photosubstitution quantum yields Φ_517_ (Table 2; Figure S5) [43]. The quantum yields of [**1**](PF_6_)_2_ and [**2**](PF_6_)_2_ were found similar (Φ_517_ = 0.022 and 0.023 for [**1**]^2+^ and [**2**]^2+^, respectively). Thus, changing the bidentate ligand from bpy to i-biq did not alter the photosubstitution efficacy. However, the presence of i-Hdiqa in [**3**]^2+^ increased the quantum yield by a 3.5-fold, to Φ_517_ = 0.077, which is quite high.

The reason for the increased photosubstitution quantum yield of the Hmte ligand in [**3**]^2+^ remains unclear. Triplet state minimization using DFT afforded, as expected, 2 different triplet states for each complex (Table S7-S12): an ^3^MLCT state, characterized by a geometry very similar to the ground state and a highest singly occupied orbital (SOMO) located on the terpyridine ligand; and a ^3^MC state, characterized by strongly elongated Ru-S and Ru-N_5_^trans^ bonds (Table 3) and a highest SOMO primarily located on the metal. The difference in energy ΔE between the ^3^MC and ^3^MLCT states (Table 3), which is often considered as a predictive parameter for photosubstitution quantum efficiencies, did not correlate with the experimentally observed photosubstitution quantum yields. For [**2**]^2+^ and [**3**]^2+^ the ^3^MLCT state was found at a serendipitously equal energy of 190 kJ.mol^−1^ above their respective ground states, which corresponds to a stabilization by 31 kJ mol^−1^, compared to [**1**]^2+^ (Table 3). However, the ^3^MC state for [**3**]^2+^ was found higher in energy (210 kJ mol^−1^) compared to that for [**2**]^2+^. The ^3^MLCT-^3^MC gap energy ΔE predicted photosubstitution quantum yields **Φ**_**517**_ to increase along the series [**3**]^2+^  < [**2**]^2+^  < [**1**]^2+^, while the experimental trend was [**1**]^2+^  ~ [**2**]^2+^  < [**3**]^2+^. On the one hand, this discrepancy goes in line with recent finding from the Turro group demonstrating that photosubstitution quantum yields in a series of related ruthenium complexes follow reverse energy gap law, and that population of ^3^MC states may not be necessary to trigger photosubstitution [44]. On the other hand, [**2**]^2+^ and [**3**]^2+^ have the same ^3^MLCT energy level, while the latter shows significantly higher photosubstitution quantum yields; other phenomena, such as interaction with the incoming solvent molecule, may also explain this discrepancy, which should be studied further. To conclude on the photochemistry, the phosphorescence of all three complexes in deuterated methanol was negligible (Φ_P_ < 5 · 10^−4^ upon blue light irradiation), while they showed very low singlet oxygen quantum yields Φ_Δ_, suggesting that their ^3^MLCT states might be short-lived, and that these complexes are not suitable for PDT (Table 2; Figure S3). Overall, photochemical generation of the ^3^MLCT state of these compounds resulted in significant ([**1**]^2+^, [**2**]^2+^) to high ([**3**]^2+^) photosubstitution quantum yields, possibly *not* via thermal population of their ^3^MC states, while their phosphorescence and singlet oxygen quantum yields remained negligible. Therefore, like [**1**]^2+^ complexes [**2**]^2+^ and [**3**]^2+^ fulfill the photochemical criteria of potential PACT candidates.

**Table 3:** Triplet state DFT calculations in water for complexes [**1**]^2+^–[**3**]^2+^^a^

Complex	NN	Type of triplet	Relative energy (kJ/mol)	ΔE ^*b*^ (kJmol^−1^)	Ru-S (Å)	Ru-N_5_^trans^ (Å)	Ru-N_4_ (Å)
[**1**]^2+^	bpy	^3^MLCT ^3^MC	221 196	− 25	2.401 3.064	2.077 2.253	2.102 2.126
[**2**]^2+^	i-biq	^3^MLCT ^3^MC	190 198	+ 8	2.403 2.941	2.086 2.360	2.102 2.142
[**3**]^2+^	i-Hdiqa	^3^MLCT ^3^MC	190 210	+ 30	2.428 3.049	2.096 2.371	2.121 2.134

^a^Level of theory: PBE0/TZP/COSMO(water)

^b^Defined as *ΔE* = *E(*^*3*^*MC)-E(*^*3*^*MLCT)*

### Cytotoxicity and cellular uptake

The thermal stability of PACT complexes is essential not only in pure water, but also in cell-growing conditions, i.e., in OptiMEM complete medium at 37 °C. All three complexes [**1**](PF_6_)_2_-[**3**](PF_6_)_2_ were found stable for at least 24 h under such conditions (Figure S1c and S1d). In a second step, the cytotoxicity of these complexes was tested under normoxic conditions (21% O_2_) in 2D monolayers of human lung carcinoma (A549) and human epidermoid carcinoma (A431) cell lines, following a protocol developed in our group [45]. In short, cancer cells were seeded at *t* = 0 h, treated with six different complex concentrations at *t* = 24 h, and irradiated after another 24 h with the light of a green LED for 30 min (520 nm, 38 J/cm^2^). The irradiation time, necessary to fully activate the complexes, was determined in a mock irradiation protocol using UV–vis spectroscopy (Figure S10). At *t* = 96 h a Sulforhodamine B (SRB) assay was performed to compare the cell viability in treated vs. untreated cells (Figure S7 and S8). The effective concentrations (EC_50_ values), i.e. the concentration at which the cell viability was reduced by 50% compared to untreated cells, are reported in Table 4. The photo index of each compound was calculated as the ratio of the EC_50_ values obtained in the dark and upon light irradiation.

**Table 4:** (Photo)cytotoxicity (EC_50_ with 95% confidence interval in µM) of [**1**](PF_6_)_2_, [**2**](PF_6_)_2_, [**3**](PF_6_)_2_, and Hmte in lung cancer cells (A549) and skin cancer cells (A431) under normoxic conditions (21% O_2_)^a^. Cellular uptake (CU in nmol Ru/mg cell protein) of [**1**](PF_6_)_2_–[**3**](PF_6_)_2_ in lung cancer cells (A549) under normoxic conditions (21%)^b^

Cell		[**1**](PF_6_)_2_	[**2**](PF_6_)_2_		[**3**](PF_6_)_2_		Hmte
A549	EC50,dark	> 150	79.7	+ 6.1 − 5.7	62.1	+ 16.4 − 13.8	> 150
	light	> 150	20.6	+ 3.0 − 2.6	13.8	+ 4.3 − 3.6	> 150
	PI^c^	–	3.9		4.5		–
	CU	0.16 ± 0.11	0.32 ± 0.14		0.69 ± 0.16		–
A431	Dark	> 150	55.2	+ 7.5 − 6.5	42.9	+ 9.2 − 7.5	> 150
	Light	> 150	12.2	+ 1.5 − 1.4	11.2	+ 2.7 − 2.4	> 150
	PI^c^	–	4.5		3.8		–

^a^Cytotoxicity experiments were performed in biological and technical triplicate; errors indicate 95% confidence intervals (in μM)

^b^Results of cellular uptake (CU) experiments upon incubation for 24 h with 30 µM drug in the dark. Value is average from a biological triplicate experiment, error is the standard deviation; ^c^photo index (PI) is defined as EC_50, dark_/EC_50, light_

The bpy-based complex [**1**](PF_6_)_2_ was found as expected to be non-cytotoxic against A549 cancer cells, whether irradiated or not (EC_50_ > 150 µM). The complexes [**2**](PF_6_)_2_ and [**3**](PF_6_)_2_ showed low cytotoxicity in the dark (80 vs. 62 µM), but revealed a significant increase in cytotoxicity after light activation characterized by EC_50_ values of 21 and 14 µM, respectively. These changes correspond to photo indices of ~ 4 for both complexes, indicating that a more cytotoxic species is released upon light activation. The released thioether ligand Hmte, tested independently, showed neither cytotoxicity in the dark nor upon light irradiation (Figure S9). In A431 cancer cells, the same trends were observed (Table 4). Therefore, the cytotoxicity observed upon light irradiation of [**2**]^2+^ or [**3**]^2+^ must be based on the metal-containing photoproduct, *i.e*. the aqua complexes [**4**]^2+^ and [**5**]^2+^, respectively, and not on the photoreleased Hmte ligand [46, 47].

To quantify the effect of the increased hydrophobicity of the complexes on the cellular uptake, uptake experiments were performed. A549 cells were treated with 30 µM of the complex [**1**](PF_6_)_2_–[**3**](PF_6_)_2_, which is lower than their dark EC_50_ values, and the ruthenium uptake was determined after 24 h incubation in the dark (Table 4). The ruthenium content in nmol Ru per mg cell protein was determined by high-resolution continuum-source atomic absorption spectrometry (HRCS AAS, further details in ESI) under normoxic (21% O_2_). It should be noted here that in such an assay, we cannot distinguish aggregation of the complexes onto the cell surface, from real internalization of the complex (i.e., by passive or active crossing of the cellular membrane): the “uptake” results actually expressed the sum of both types of molecules. Complex [**1**](PF_6_)_2_ was less taken up (0.16 nmol per mg cell protein), compared to the other two complexes [**2**](PF_6_)_2_ and [**3**](PF_6_)_2_, for which the ruthenium uptake was 0.32 and 0.69 nmol per mg cell protein, respectively. Probably, the higher lipophilicity of [**2**](PF_6_)_2_ and [**3**](PF_6_)_2_, compared to their bpy analog, is at least partly responsible for their higher uptake. However, the more polar (log P_ow_ = 0.45) i-Hdiqa complex [**3**]^2+^ showed enhanced accumulation, compared to its more hydrophobic i-biq analog [**2**]^2+^ (log P_ow_ = 2.10), so that some active transport may be involved here.

Increased uptake with polypyridyl ligands bearing a non-coordinating secondary amine group has been observed for example by Barton et al. with rhodium(III) complexes [29], or in our group by platinum(II) complexes [48]; however, the reason for such phenomenon in ruthenium(II) complexes remain unclear. Next to hypothesizing some form of active transport, we may also speculate that metal complexes bearing non-coordinated NH groups such as [**3**]^2+^, may partly be deprotonated because of the increased acidity of the NH group upon metal coordination, which may lower the charge of the metal complex and hence improve cellular uptake by passive diffusion. For example, a concentration-dependent pKa value between 4 and 5 was reported in acetonitrile for [Ru(phen)_2_(HDPA)]^2+^ (phen = 1,10-phenanthroline, HDPA = 2,2’-dipyridylamine) [49]. We are unaware of similar pKa measurements in aqueous solution for ruthenium(II)-dipyridylamine complexes. We should also mention that for the platinum(II) complex [Pt(H_2_bapbpy)]^2+^ (where H_2_bapbpy = is N-(6-(6-(pyridin-2-ylamino)- pyridin-2-yl)pyridin-2-yl)pyridin-2-amine), a pKa of 8.3 was measured in water, which was accompanied by a massive cellular uptake in A549 lung cancer cells (1586 pmol Pt/million cells), compared to cisplatin (23 pmol Pt/million cells). However, we did not notice during our investigations on [**3**]^2+^, any sign of deprotonation in aqueous solution near pH = 7.4, so that such arguments remain, at that moment, pure speculation. Another hypothesis is that hydrogen bonding involving the non-coordinated NH bridge and biological anions would lead to better transport of the complex through the cell membrane [50]. All in all, the difference in cellular toxicity between [**1**](PF_6_)_2_ on the one hand, and [**2**](PF_6_)_2_ and [**3**](PF_6_)_2_ on the other hand, probably come from other reasons than differences in cellular uptake. Clear differences of localization and/or toxicity have been observed in other published series of ruthenium polypyridyl complexes containing one or several dipyridylamine ancillary ligands [51, 52]. Probably, [Ru(tpy)(bpy)(OH_2_)]^2+^ is simply less cytotoxic than its i-biq and i-Hdiqa analogs [**4**]^2+^ and [**5**]^2+^, because of different cellular localization and/or interaction with biomolecules, which remains to be elucidated.

## Conclusions

The chemically photoactivatable ruthenium complex [**1**](PF_6_)_2_ is poorly taken up by cells and showed no (photo)cytotoxicity in cancer cells. Therefore, although it is chemically activated by light it is not biologically activated by light in cells, and hence not suitable as a PACT agent. However, two analog ruthenium complexes with more hydrophobic bidentate ligands were shown to be promising PACT compounds. [**2**](PF_6_)_2_ showed a photosubstitution quantum yield that was comparable with that of [**1**](PF_6_)_2_ and a higher cellular uptake, overall resulting in increased cytotoxicity upon green light irradiation. [**3**](PF_6_)_2_, which has an additional non-coordinated amine bridge, showed enhanced photosubstitution quantum yield compared to [**2**]^2+^ and the highest cellular uptake in the series, but its photoindex was similar, in the tested conditions, to that of [**2**]^2+^. This work demonstrates that careful considerations on ligand design are necessary to fine-tune light activation of a Ru-based PACT drug. The lipophilicity of the prodrug, which influences cellular uptake and interaction with biomolecules, must be intermediate, and its ligand exchange properties must be slow in the dark and significantly increased upon visible light irradiation.

## Supplementary Information

Below is the link to the electronic supplementary material.

Supplementary file1 (ZIP 17 KB)

Supplementary file2 (CIF 927 KB)

Supplementary file3 (PDF 231 KB)

Supplementary file3 (PDF 5297 KB)

## References

[1] Zayat L, Filevich O, Baraldo LM, Etchenique R (2013) Philos Trans Royal Soc A Math Phys Eng Sci 371:20120330 10.1098/rsta.2012.0330 23776299

[2] Battistin F, Balducci G, Wei J, Renfrew AK, Alessio E (2018) Eur J Inorg Chem 2018:1469–1480

[3] Ragazzon G, Bratsos I, Alessio E, Salassa L, Habtemariam A, McQuitty RJ, Clarkson GJ, Sadler PJ (2012) Inorg Chim Acta 393:230–238

[4] Zayat L, Calero C, Alborés P, Baraldo L, Etchenique R (2003) J Am Chem Soc 125:882–883 12537482 10.1021/ja0278943

[5] Sun W, Li S, Häupler B, Liu J, Jin S, Steffen W, Schubert US, Butt H-J, Liang X-J, Wu S (2017) Adv Mater 29:1603702 10.1002/adma.201603702 27918115

[6] Li A, Turro C, Kodanko JJ (2018) Acc Chem Res 51:1415–1421 29870227 10.1021/acs.accounts.8b00066 PMC6019290

[7] Li A, Turro C, Kodanko JJ (2018) Chem Commun 54:1280–1290 10.1039/c7cc09000e PMC5904840 29323683

[8] Havrylyuk D, Heidary DK, Sun Y, Parkin S, Glazer EC (2020) ACS Omega 5:18894–18906 32775891 10.1021/acsomega.0c02079 PMC7408248

[9] Havrylyuk D, Stevens K, Parkin S, Glazer EC (2020) Inorg Chem 59:1006–1013 31899619 10.1021/acs.inorgchem.9b02065 PMC8607748

[10] Roque Iii J, Havrylyuk D, Barrett PC, Sainuddin T, McCain J, Colón K, Sparks WT, Bradner E, Monro S, Heidary D, Cameron CG, Glazer EC, McFarland SA (2020) Photochem Photobiol 96:327–339 31691282 10.1111/php.13174 PMC7138741

[11] Garner RN, Gallucci JC, Dunbar KR, Turro C (2011) Inorg Chem 50:9213–9215 21879748 10.1021/ic201615u PMC4556276

[12] Al-Afyouni MH, Rohrabaugh TN, Al-Afyouni KF, Turro C (2018) Chem Sci 9:6711–6720 30310605 10.1039/c8sc02094a PMC6115629

[13] Wei J, Renfrew AK (2018) J Inorg Biochem 179:146–153 29180165 10.1016/j.jinorgbio.2017.11.018

[14] Chan H, Ghrayche JB, Wei J, Renfrew AK (2017) Eur J Inorg Chem 2017:1679–1686

[15] Basu U, Karges J, Chotard F, Balan C, Le Gendre P, Gasser G, Bodio E, Malacea Kabbara R (2019) Polyhedron 172:22–27

[16] Sun W, Wen Y, Thiramanas R, Chen M, Han J, Gong N, Wagner M, Jiang S, Meijer MS, Bonnet S, Butt H-J, Mailänder V, Liang X-J, Wu S (2018) Adv Func Mater 28:1804227

[17] Lameijer LN, Brevé TG, van Rixel VHS, Askes SHC, Siegler MA, Bonnet S (2018) Chem A Eur J 24:2709–2717 10.1002/chem.201705388 PMC5838788 29220545

[18] Bahreman A, Limburg B, Siegler MA, Bouwman E, Bonnet S (2013) Inorg Chem 52:9456–9469 23909908 10.1021/ic401105v

[19] Novakova O, Kasparkova J, Vrana O, van Vliet PM, Reedijk J, Brabec V (1995) Biochemistry 34:12369–12378 7547981 10.1021/bi00038a034

[20] Alessio E (2017) Eur J Inorg Chem 2017:1549–1560

[21] Lameijer LN, van de Griend C, Hopkins SL, Volbeda A-G, Askes SHC, Siegler MA, Bonnet S (2019) J Am Chem Soc 141:352–362 30525567 10.1021/jacs.8b10264 PMC6331141

[22] Huang H, Zhang P, Chen H, Ji L, Chao H (2015) Chem A Eur J 21:715–725 10.1002/chem.201404922 25388328

[23] Schatzschneider U, Niesel J, Ott I, Gust R, Alborzinia H, Wölfl S (2008) ChemMedChem 3:1104–1109 18425738 10.1002/cmdc.200800039

[24] Cuello-Garibo J-A, James CC, Siegler MA, Bonnet S (2017) Chem Sq 1:1–19

[25] Siewert B, van Rixel VH, van Rooden EJ, Hopkins SL, Moester MJ, Ariese F, Siegler MA, Bonnet S (2016) Chem Eur J 22:10960–10968 27373895 10.1002/chem.201600927 PMC5096026

[26] Howerton BS, Heidary DK, Glazer EC (2012) J Am Chem Soc 134:8324–8327 22553960 10.1021/ja3009677

[27] Kohler L, Nease L, Vo P, Garofolo J, Heidary DK, Thummel RP, Glazer EC (2017) Inorg Chem 56:12214–12223 28949518 10.1021/acs.inorgchem.7b01642 PMC6040668

[28] Lameijer LN, Ernst D, Hopkins SL, Meijer MS, Askes SH, Le Dévédec SE, Bonnet S (2017) Angew Chem Int Ed 56:11549–11553 10.1002/anie.201703890 PMC5601216 28666065

[29] Komor AC, Schneider CJ, Weidmann AG, Barton JK (2012) J Am Chem Soc 134:19223–19233 23137296 10.1021/ja3090687 PMC3740518

[30] van Rixel VHS, Siewert B, Hopkins SL, Askes SHC, Busemann A, Siegler MA, Bonnet S (2016) Chem Sci 7:4922–4929 30155140 10.1039/c6sc00167j PMC6018302

[31] Han Ang W, Dyson PJ (2006) Eur J Inorg Chem 2006:4003–4018

[32] Funayama T, Kato M, Kosugi H, Yagi M, Higuchi J, Yamauchi S (2000) Bull Chem Soc Jpn 73:1541–1550

[33] Hou Z, Nishiura M, Rai VK (2011) Novel Complex Compound and Use Thereof. WO2012114940A1, application date 2012/08/30/, application number JP2012053392W, priority numbers JP2011038483A·2011-02-24

[34] Marion R, Sguerra F, Di Meo F, Sauvageot E, Lohier J-F, Daniellou R, Renaud J-L, Linares M, Hamel M, Gaillard S (2014) Inorg Chem 53:9181–9191 25134011 10.1021/ic501230m

[35] Toyama M, Suganoya R, Tsuduura D, Nagao N (2007) Bull Chem Soc Jpn 80:922–936

[36] Chanda N, Mobin SM, Puranik VG, Datta A, Niemeyer M, Lahiri GK (2004) Inorg Chem 43:1056–1064 14753828 10.1021/ic034902n

[37] Robinson K, Gibbs GV, Ribbe PH (1971) Science 172:567–570 17802221 10.1126/science.172.3983.567

[38] Fleet ME (1976) Mineral Mag 40:531–533

[39] Garcìa-Fresnadillo D, Georgiadou Y, Orellana G, Braun AM, Oliveros E (1996) Helv Chim Acta 79:1222–1238

[40] Busemann A, Araman C, Flaspohler I, Pratesi A, Zhou X-Q, van Rixel VHS, Siegler MA, Messori L, van Kasteren SI, Bonnet S (2020) Inorg Chem 59:7710–7720 32396371 10.1021/acs.inorgchem.0c00742 PMC7268191

[41] Goldbach RE, Rodriguez-Garcia I, van Lenthe JH, Siegler MA, Bonnet S (2011) Chem Eur J 17:9924–9929 21796695 10.1002/chem.201101541

[42] Siewert B, Langerman M, Hontani Y, Kennis JTM, van Rixel VHS, Limburg B, Siegler MA, Talens Saez V, Kieltyka RE, Bonnet S (2017) Chem Commun 53:11126–11129 10.1039/c7cc02989f 28682371

[43] Snellenburg JJ, Laptenok SP, Seger R, Mullen KM, Van Stokkum IH (2012) J Stat Softw 49:1–22

[44] Loftus LM, Rack JJ, Turro C (2020) Chem Commun 56:4070–4073 10.1039/c9cc10095d 32159547

[45] Hopkins S, Siewert B, Askes S, Veldhuizen P, Zwier R, Heger M, Bonnet S (2016) Photochem Photobiol Sci 15:644–653 27098927 10.1039/c5pp00424a PMC5044800

[46] Cuello-Garibo J-A, Meijer MS, Bonnet S (2017) Chem Commun 53:6768–6771 10.1039/c7cc03469e PMC5708332 28597879

[47] Azar DF, Audi H, Farhat S, El-Sibai M, Abi-Habib RJ, Khnayzer RS (2017) Dalton Trans 46:11529–11532 28748239 10.1039/c7dt02255g

[48] van Rixel VHS, Busemann A, Wissingh MF, Hopkins SL, Siewert B, van de Griend C, Siegler MA, Marzo T, Papi F, Ferraroni M, Gratteri P, Bazzicalupi C, Messori L, Bonnet S (2019) Angew Chem Int Ed 58:9378–9382 10.1002/anie.201814532 PMC6618160 31046177

[49] Drew MGB, Nag S, Datta D (2008) Inorg Chim Acta 361:417–421

[50] Patil SK, Ghosh R, Kennedy P, Mobin SM, Das D (2016) RSC Adv 6:62310–62319

[51] Putta VR, Chintakuntla N, Mallepally RR, Avudoddi S, Nancherla NKD, Surya SS, Sirasani S (2016) J Fluoresc 26:225–240 26555289 10.1007/s10895-015-1705-z

[52] Mari C, Pierroz V, Leonidova A, Ferrari S, Gasser G (2015) Eur J Inorg Chem 2015:3879–3891 10.1021/acs.inorgchem.5b01332 26440628

