# Supplementary material for: Ruthenium-based PACT agents based on bisquinoline chelates: synthesis, photochemistry, and cytotoxicity
Source: J Biol Inorg Chem. 2021 Aug 10;26(6):667–74. doi: 10.1007/s00775-021-01882-8 (PMC8437835; doi:10.1007/s00775-021-01882-8)

checkCIF/PLATON report

Structure factors have been supplied for datablock(s) xs1446a

THIS REPORT IS FOR GUIDANCE ONLY. IF USED AS PART OF A REVIEW PROCEDURE FOR PUBLICATION, IT SHOULD NOT REPLACE THE EXPERTISE OF AN EXPERIENCED CRYSTALLOGRAPHIC REFEREE.

No syntax errors found. CIF dictionary Interpreting this report

Datablock: xs1446a

```
Bond precision:  C-C = 0.0172 A          Wavelength=1.54178

Cell:            a=22.0959(11)      b=8.8289(2)     c=37.3521(9)
                 alpha=90           beta=90         gamma=90
Temperature:     110 K

                Calculated                 Reported
Volume          7286.7(4)                  7286.7(4)
Space group     P c a 21                   P c a 21
Hall group      P 2c -2ac                  P 2c -2ac
Moiety formula  C36 H30 N5 O Ru S, 2(F6 P) C36 H30 N5 O Ru S, 2(F6 P)
Sum formula     C36 H30 F12 N5 O P2 Ru S   C36 H30 F12 N5 O P2 Ru S
Mr              971.72                     971.72
Dx,g cm-3       1.772                      1.772
Z               8                          8
Mu (mm-1)       5.778                      5.778
F000            3896.0                     3896.0
F000'           3917.36
h,k,lmax        27,10,46                   27,10,46
Nref            14308[ 7272]               11592
Tmin,Tmax       0.525,0.749                0.280,0.812
Tmin'           0.050

Correction method= # Reported T Limits: Tmin=0.280 Tmax=0.812
AbsCorr = ANALYTICAL

Data completeness= 1.59/0.81      Theta(max)= 71.875

R(reflections)= 0.0525( 10905)    wR2(reflections)= 0.1407( 11592)

S = 1.109                  Npar= 1423
```

The following ALERTS were generated. Each ALERT has the format
test-name_ALERT_alert-type_alert-level.
Click on the hyperlinks for more details of the test.

Alert level B

```
PLAT090_ALERT_3_B Poor Data / Parameter Ratio (Zmax > 18) ........       5.05 Note
```

Alert level C

```
STRVA01_ALERT_4_C          Flack test results are ambiguous.
           From the CIF: _refine_ls_abs_structure_Flack    0.539
           From the CIF: _refine_ls_abs_structure_Flack_su    0.016
PLAT234_ALERT_4_C Large Hirshfeld Difference O1A      --C36A     .       0.16 Ang.
PLAT234_ALERT_4_C Large Hirshfeld Difference C24A     --C25A     .       0.16 Ang.
PLAT234_ALERT_4_C Large Hirshfeld Difference P3       --F15      .       0.17 Ang.
PLAT234_ALERT_4_C Large Hirshfeld Difference P3       --F17      .       0.16 Ang.
PLAT250_ALERT_2_C Large U3/U1 Ratio for Average U(i,j) Tensor ....        2.2 Note
PLAT250_ALERT_2_C Large U3/U1 Ratio for Average U(i,j) Tensor ....        2.2 Note
PLAT309_ALERT_2_C Single Bonded Oxygen (C-O > 1.3 Ang) ..........       O1A' Check
PLAT309_ALERT_2_C Single Bonded Oxygen (C-O > 1.3 Ang) ..........        O1A Check
PLAT342_ALERT_3_C Low Bond Precision on  C-C Bonds ...............    0.01719 Ang.
PLAT907_ALERT_2_C Flack x > 0.5, Structure Needs to be Inverted? .       0.54 Check
PLAT911_ALERT_3_C Missing FCF Refl Between Thmin & STh/L=    0.600         78 Report
PLAT921_ALERT_1_C R1  in the CIF and FCF Differ by ..............     0.0011 Check
PLAT922_ALERT_1_C wR2 in the CIF and FCF Differ by ..............     0.0020 Check
PLAT923_ALERT_1_C S    Values in the CIF and FCF Differ by .......      0.016 Check
PLAT971_ALERT_2_C Check Calcd Resid. Dens.  0.86A   From Ru1             1.82 eA-3
PLAT971_ALERT_2_C Check Calcd Resid. Dens.  0.64A   From Ru2             1.63 eA-3
```

Alert level G

```
PLAT002_ALERT_2_G Number of Distance or Angle Restraints on AtSite         94 Note
PLAT003_ALERT_2_G Number of Uiso or Uij Restrained non-H Atoms ...         95 Report
PLAT083_ALERT_2_G SHELXL Second Parameter in WGHT  Unusually Large      44.23 Why ?
PLAT168_ALERT_4_G The CIF-Embedded .res File Contains EXYZ Records          1 Report
PLAT171_ALERT_4_G The CIF-Embedded .res File Contains EADP Records         10 Report
PLAT175_ALERT_4_G The CIF-Embedded .res File Contains SAME Records          8 Report
PLAT176_ALERT_4_G The CIF-Embedded .res File Contains SADI Records          5 Report
PLAT178_ALERT_4_G The CIF-Embedded .res File Contains SIMU Records          5 Report
PLAT187_ALERT_4_G The CIF-Embedded .res File Contains RIGU Records          7 Report
PLAT244_ALERT_4_G Low    'Solvent' Ueq as Compared to Neighbors of         P1 Check
PLAT244_ALERT_4_G Low    'Solvent' Ueq as Compared to Neighbors of         P2 Check
PLAT301_ALERT_3_G Main Residue  Disorder .............(Resd  1  )         45% Note
PLAT301_ALERT_3_G Main Residue  Disorder .............(Resd  2  )          2% Note
PLAT302_ALERT_4_G Anion/Solvent/Minor-Residue Disorder (Resd  5  )       100% Note
PLAT302_ALERT_4_G Anion/Solvent/Minor-Residue Disorder (Resd  6  )       100% Note
PLAT302_ALERT_4_G Anion/Solvent/Minor-Residue Disorder (Resd  7  )       100% Note
PLAT302_ALERT_4_G Anion/Solvent/Minor-Residue Disorder (Resd  8  )       100% Note
PLAT302_ALERT_4_G Anion/Solvent/Minor-Residue Disorder (Resd  9  )       100% Note
PLAT302_ALERT_4_G Anion/Solvent/Minor-Residue Disorder (Resd 10  )       100% Note
PLAT304_ALERT_4_G Non-Integer Number of Atoms in ..... (Resd  5  )       4.31 Check
PLAT304_ALERT_4_G Non-Integer Number of Atoms in ..... (Resd  6  )       1.39 Check
PLAT304_ALERT_4_G Non-Integer Number of Atoms in ..... (Resd  7  )       1.30 Check
PLAT304_ALERT_4_G Non-Integer Number of Atoms in ..... (Resd  8  )       2.51 Check
PLAT304_ALERT_4_G Non-Integer Number of Atoms in ..... (Resd  9  )       2.39 Check
PLAT304_ALERT_4_G Non-Integer Number of Atoms in ..... (Resd 10  )       2.10 Check
PLAT432_ALERT_2_G Short Inter X...Y Contact  F4       ..C4A              2.90 Ang.
                                               -1/2+x,2-y,z  =      3_475 Check
PLAT432_ALERT_2_G Short Inter X...Y Contact  F13C     ..C35A             2.69 Ang.
                                                      x,y,z  =      1_555 Check
PLAT432_ALERT_2_G Short Inter X...Y Contact  F13C     ..C36A             2.72 Ang.
                                                      x,y,z  =      1_555 Check
PLAT432_ALERT_2_G Short Inter X...Y Contact  F14C     ..C13A             2.83 Ang.
                                                    x,1+y,z  =      1_565 Check
PLAT432_ALERT_2_G Short Inter X...Y Contact  F15'     ..C3A              2.90 Ang.
```

```
                                              -1/2+x,2-y,z  =      3_475 Check
PLAT432_ALERT_2_G Short Inter X...Y Contact  F15'     ..C2A             2.97 Ang.
                                              -1/2+x,2-y,z  =      3_475 Check
PLAT432_ALERT_2_G Short Inter X...Y Contact  F19'     ..C9A             2.94 Ang.
                                             1-x,1-y,1/2+z  =      2_665 Check
PLAT720_ALERT_4_G Number of Unusual/Non-Standard Labels ..........          2 Note
PLAT790_ALERT_4_G Centre of Gravity not Within Unit Cell: Resd.  #          3 Note
              F6 P
PLAT790_ALERT_4_G Centre of Gravity not Within Unit Cell: Resd.  #          4 Note
              F6 P
PLAT794_ALERT_5_G Tentative Bond Valency for Ru1       (III)     .       3.18 Info
PLAT811_ALERT_5_G No ADDSYM Analysis: Too Many Excluded Atoms ....          ! Info
PLAT860_ALERT_3_G Number of Least-Squares Restraints .............       3040 Note
PLAT910_ALERT_3_G Missing # of FCF Reflection(s) Below Theta(Min).          1 Note
PLAT912_ALERT_4_G Missing # of FCF Reflections Above STh/L=  0.600          8 Note
PLAT915_ALERT_3_G No Flack x Check Done: Low Friedel Pair Coverage        63 %
PLAT933_ALERT_2_G Number of OMIT Records in Embedded .res File ...          6 Note
PLAT941_ALERT_3_G Average HKL Measurement Multiplicity ...........        3.3 Low
PLAT965_ALERT_2_G The SHELXL WEIGHT Optimisation has not Converged     Please Check
PLAT978_ALERT_2_G Number C-C Bonds with Positive Residual Density.          0 Info
```

```
   0 ALERT level A = Most likely a serious problem - resolve or explain
   1 ALERT level B = A potentially serious problem, consider carefully
  17 ALERT level C = Check. Ensure it is not caused by an omission or oversight
  45 ALERT level G = General information/check it is not something unexpected

   3 ALERT type 1 CIF construction/syntax error, inconsistent or missing data
  20 ALERT type 2 Indicator that the structure model may be wrong or deficient
   9 ALERT type 3 Indicator that the structure quality may be low
  29 ALERT type 4 Improvement, methodology, query or suggestion
   2 ALERT type 5 Informative message, check
```

Validation response form

Please find below a validation response form (VRF) that can be filled in and pasted into your CIF.

```
# start Validation Reply Form
_vrf_STRVA01_xs1446a
;
PROBLEM: Flack test results are ambiguous.
RESPONSE: ...
;
_vrf_PLAT090_xs1446a
;
PROBLEM: Poor Data / Parameter Ratio (Zmax > 18) ........       5.05 Note
RESPONSE: ...
;
_vrf_PLAT234_xs1446a
;
PROBLEM: Large Hirshfeld Difference O1A      --C36A     .       0.16 Ang.
RESPONSE: ...
;
_vrf_PLAT250_xs1446a
;
PROBLEM: Large U3/U1 Ratio for Average U(i,j) Tensor ....        2.2 Note
RESPONSE: ...
;
_vrf_PLAT309_xs1446a
;
```

```
PROBLEM: Single Bonded Oxygen (C-O > 1.3 Ang) ...........       O1A’ Check
RESPONSE: ...
;
_vrf_PLAT342_xs1446a
;
PROBLEM: Low Bond Precision on  C-C Bonds ...............    0.01719 Ang.
RESPONSE: ...
;
_vrf_PLAT907_xs1446a
;
PROBLEM: Flack x > 0.5, Structure Needs to be Inverted? .       0.54 Check
RESPONSE: ...
;
_vrf_PLAT911_xs1446a
;
PROBLEM: Missing FCF Refl Between Thmin & STh/L=    0.600         78 Report
RESPONSE: ...
;
_vrf_PLAT921_xs1446a
;
PROBLEM: R1  in the CIF and FCF Differ by ...............     0.0011 Check
RESPONSE: ...
;
_vrf_PLAT922_xs1446a
;
PROBLEM: wR2 in the CIF and FCF Differ by ...............     0.0020 Check
RESPONSE: ...
;
_vrf_PLAT923_xs1446a
;
PROBLEM: S    Values in the CIF and FCF Differ by .......      0.016 Check
RESPONSE: ...
;
_vrf_PLAT971_xs1446a
;
PROBLEM: Check Calcd Resid. Dens.  0.86A   From Ru1             1.82 eA-3
RESPONSE: ...
;
# end Validation Reply Form
```

It is advisable to attempt to resolve as many as possible of the alerts in all categories. Often the minor alerts point to easily fixed oversights, errors and omissions in your CIF or refinement strategy, so attention to these fine details can be worthwhile. In order to resolve some of the more serious problems it may be necessary to carry out additional measurements or structure refinements. However, the purpose of your study may justify the reported deviations and the more serious of these should normally be commented upon in the discussion or experimental section of a paper or in the "special_details" fields of the CIF. checkCIF was carefully designed to identify outliers and unusual parameters, but every test has its limitations and alerts that are not important in a particular case may appear. Conversely, the absence of alerts does not guarantee there are no aspects of the results needing attention. It is up to the individual to critically assess their own results and, if necessary, seek expert advice.

Publication of your CIF in IUCr journals

A basic structural check has been run on your CIF. These basic checks will be run on all CIFs submitted for publication in IUCr journals (*Acta Crystallographica*, *Journal of Applied Crystallography*, *Journal of Synchrotron Radiation*); however, if you intend to submit to *Acta Crystallographica Section C* or *E* or *IUCrData*, you should make sure that full publication checks are run on the final version of your CIF prior to submission.

Publication of your CIF in other journals

Please refer to the *Notes for Authors* of the relevant journal for any special instructions relating to CIF submission.

PLATON version of 05/12/2020; check.def file version of 05/12/2020

Datablock xs1446a - ellipsoid plot

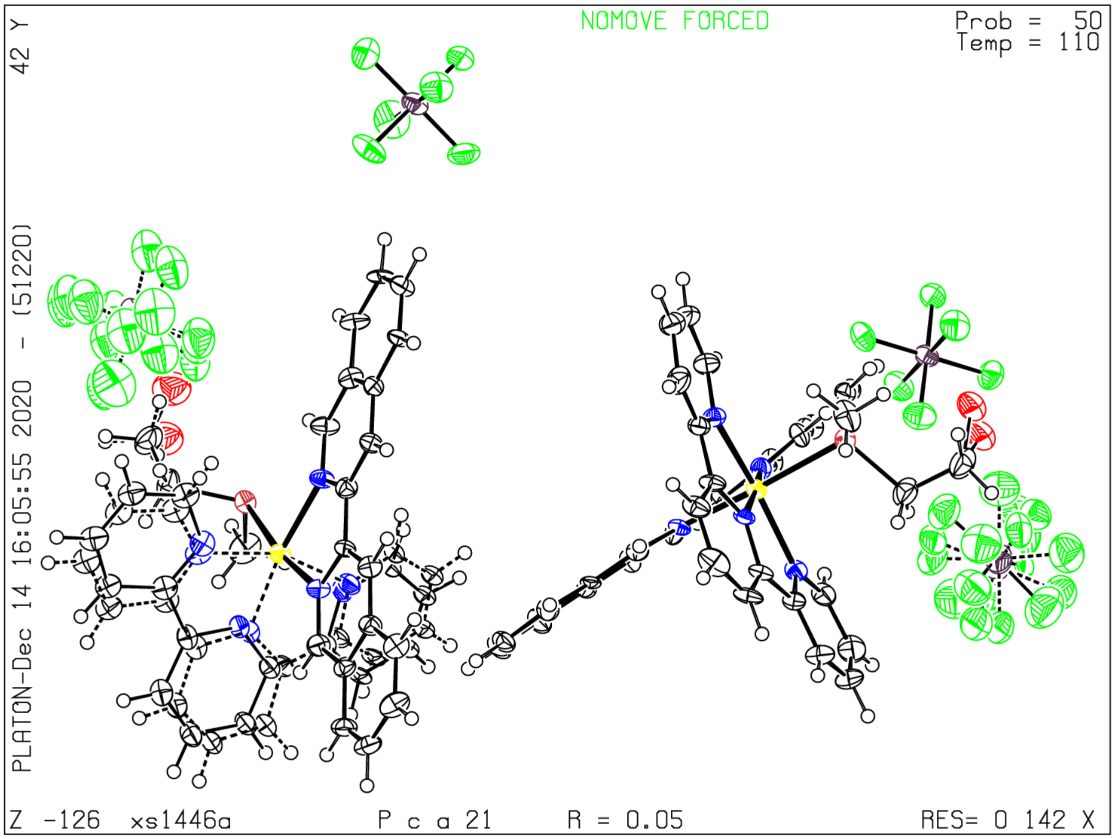

Supplement: Supplementary file 3 — Supplementary file3 (PDF 231 KB) [file 775_2021_1882_MOESM3_ESM.pdf]
