# Supplementary material for: Ruthenium-based PACT agents based on bisquinoline chelates: synthesis, photochemistry, and cytotoxicity
Source: J Biol Inorg Chem. 2021 Aug 10;26(6):667–74. doi: 10.1007/s00775-021-01882-8 (PMC8437835; doi:10.1007/s00775-021-01882-8)
Supplement: Supplementary file 4 — Supplementary file3 (PDF 5297 KB) [file 775_2021_1882_MOESM4_ESM.pdf]

Supporting information

Ruthenium-based PACT agents based on bisquinoline chelates: synthesis, photochemistry, and cytotoxicity

Anja Busemann,[1] Ingrid Flaspohler,[1] Xue-Quan Zhou,[1] Claudia Schmidt,[2] Sina K. Goetzfried,[1] Vincent H. S. van Rixel,[1] Ingo Ott,[2] Maxime A. Siegler,[3] Sylvestre Bonnet[1,*]

Table of content

Table of content 1
1. Synthesis 2
2. Single Crystal X-Ray crystallography 6
3. Log P determination by ICP-MS 7
4. Dark stability in water and OptiMEM 9
5. Molar extinction coefficient in water 10
6. Singlet oxygen production and phosphorescence 10
7. Irradiation experiments monitored with UV-vis and MS 11
8. MS of the ruthenium species after green light irradiation 11
9. Photosubstitution quantum yield simulated by Glotaran 12
10. Cell culture and EC50 (photo)cytotoxicity studies 14
11. Green light irradiation in the cell irradiation setup 17
12. Cellular uptake 18
13. DFT and TDDFT calculations 19
14. References 21

1. Synthesis

Methods and Materials

$RuCl_3$ was purchased from Alfa Aesar, 3-bromoisoquinoline from ABCR, isoquinolin-3-amine, tris(dibenzylideneacetone)dipalladium(0), 1,3-bis(diphenylphosphino)propane, and 2-(methylthio)ethanol from Sigma Aldrich, and potassium tert-butoxide from Acros Organics. [**1**]$(PF_6)_2$ was synthesized according to literature.[1] All metal complexes were synthesized in dim light and stored in darkness. All reactants and solvents were used without further purification. ^1H NMR spectra were recorded on a Bruker AV-300 spectrometer. Chemical shifts are indicated in ppm. Mass spectra were recorded by using an MSQ Plus Spectrometer.

Synthetic route

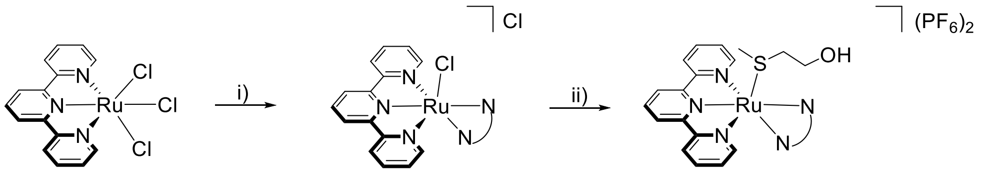

Scheme S1. Route for the synthesis of [**2**]$(PF_6)_2$ and [**3**]$(PF_6)_2$. Conditions: (i) LiCl, Et_3N, ethanol/water (3:1), N_2, reflux, i-biq (overnight, 94%) or i-Hdiqa (4 h, 83%); (ii) Hmte, $AgPF_6$, water, N_2, reflux, 4 h for [**2**]$(PF_6)_2$ (48%) and 3 h for [**3**]$(PF_6)_2$ (60%).

3,3'-biisoquinoline (i-biq)

i-biq was synthesized according to literature.[2]

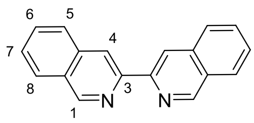

^1H NMR (300 MHz, chloroform-d, 298 K) δ 9.38 (s, 2H, 1), 8.93 (s, 2H, 4), 8.08 – 7.96 (m, 4H, 8 + 5), 7.74 (ddd, *J* = 8.2, 6.9, 1.3 Hz, 2H, 6), 7.63 (ddd, *J* = 8.1, 6.9, 1.2 Hz, 2H, 7). *^{13}C NMR (75 MHz, chloroform-d, 298 K)* δ 152.3 (1), 137.0 (3), 131.0 (6), 128.7 + 127.9 (4a + 8a), 127.9 + 127.8 (5 + 8), 127.8 (7), 118.1 (4). *ES MS m/z (calc.):* 257.3 (257.1 $[M + H]^+$).

di(isoquinolin-3-yl)amine (i-Hdiqa)

i-Hdiqa was synthesized according to literature procedures described for the synthesis of other dipyridylamine derivatives.[3]

Tris(dibenzylideneacetone)dipalladium(0) (18 mg, 0.020 mmol) and 1,3-bis(diphenylphosphino)propane (16 mg, 0.039 mmol) were dissolved in dry toluene (25 mL). 3-Bromoisoquinoline (200 mg, 0.97 mmol), isoquinolin-3-amine (170 mg, 1.2 mmol), and potassium tert-butoxide (150 mg, 1.4 mmol) were added in this order under dinitrogen atmosphere. The resultant mixture was stirred and heated to reflux under dinitrogen atmosphere overnight at 110 °C. The solution was cooled down to room temperature and filtered over *Celite*. The cake was washed four times with ethyl acetate (30 mL). The solvent was evaporated with a rotary evaporator using a water bath set at 40 °C. The crude product was purified by

column chromatography on silica with pentane/ethyl acetate 1:1 + 0.5% triethylamine as eluent (R_f = 0.75), to yield i-Hdiqa as a yellow powder. Yield: 48% (130 mg, 0.48 mmol).

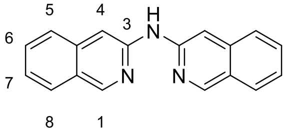

^{1}H NMR (300 MHz, chloroform-d, 298 K) δ (ppm) 9.05 (s, 2H, 1), 7.88 (dd, *J* = 8.2, 1.1 Hz, 2H, 8), 7.80 (s, 2H, 4), 7.73 (dd, *J* = 8.3, 1.1 Hz, 2H, 5), 7.64 (s, 1H, NH), 7.58 (ddd, *J* = 8.2, 6.8, 1.2 Hz, 2H, 7), 7.37 (ddd, *J* = 8.1, 6.8, 1.1 Hz, 2H, 6). *^{13}C NMR (75 MHz, chloroform-d, 298 K)* δ (ppm) 151.6 (1), 150.0 (3), 138.6 (4a), 130.7 (6), 127.9 (8), 125.8 (5), 125.2 (8a), 124.4 (7), 103.0 (4). *ES MS m/z (calc.):* 272.4 (272.1, [M + H]$^{+}$).

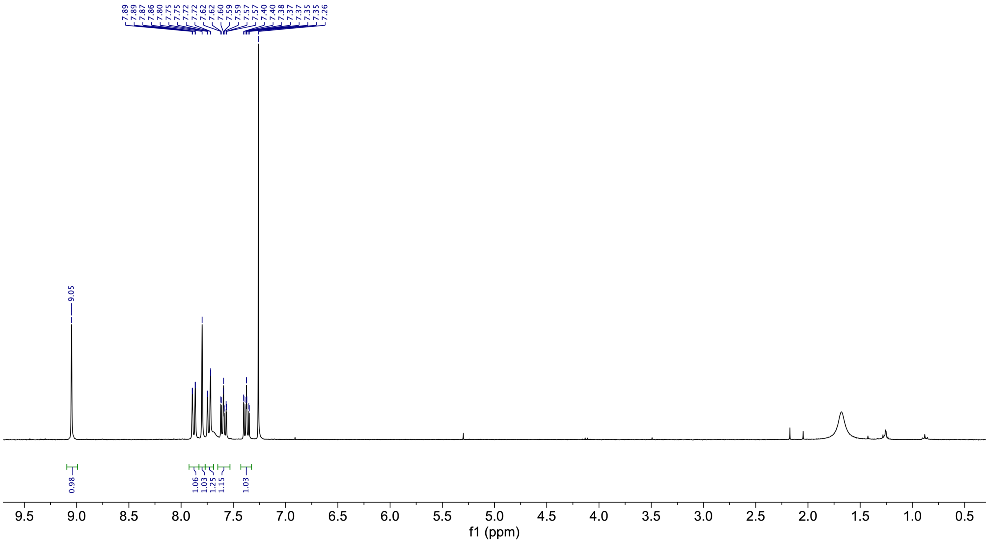

^{1}H NMR spectrum of i-Hdiqa in $CDCl_3$.

[Ru(tpy)(i-biq)(Cl)]Cl

[Ru(tpy)$(Cl)_3$] (174 mg, 0.394 mmol), i-biq (101 mg, 0.394 mmol), and lithium chloride (18.4 mg, 0.433 mmol) were dissolved in a degassed ethanol/water mixture (3:1, 32 mL). Triethylamine (0.756 mL, 0.630 mmol) was added and the reaction mixture was refluxed under dinitrogen atmosphere overnight. The reaction mixture was filtered hot over *Celite* and the cake was washed with ethanol until the filtrate was colorless. After evaporation of the solvents, the crude product was purified by column chromatography on silica with dichloromethane/methanol (9:1) as eluent (R_f = 0.64). The product was obtained as a dark brown solid. Yield: 94% (245 mg, 0.370 mmol).

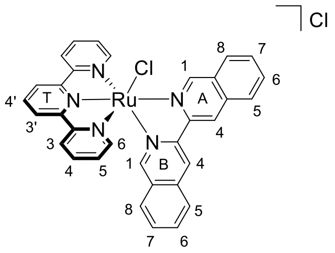

^{1}H NMR (300 MHz, methanol-d_4, 298 K) δ 10.79 (s, 1H, A1), 9.32 (s, 1H, A4), 9.03 (s, 1H, B4), 8.69 (d, *J* = 8.1 Hz, 2H, T3′), 8.55 (dt, *J* = 8.1, 1.2 Hz, 2H, T3), 8.44 – 8.33 (m, 2H, A5 + A8), 8.20 (t, *J* = 8.1 Hz, 1H, T4′), 8.10 – 7.82 (m, 8H, B5 + A6 + A7 + B1 + T6 + T4), 7.72 (ddd, *J* = 8.2, 6.5, 1.6 Hz, 1H, B6), 7.66 – 7.50 (m, 2H, B7 + B8), 7.28 (ddd, *J* = 7.3, 5.6, 1.4 Hz, 2H, T5). *^{13}C NMR (75 MHz, methanol-d_4, 298 K)* δ 160.5 + 160.0 (C_q T2 + T2′), 156.9 (A1), 156.0 (B1), 153.1 (T6), 152.6 + 151.1 (C_q A3 + B3), 138.3 (T4), 136.8 + 135.7 (C_q A4a + B4a), 135.3 (T4′), 133.7 (A6), 133.4 (B6), 131.3 (A7), 131.0 + 130.3 (C_q A8a + B8a), 130.8 (B7), 129.0 + 128.7 + 128.5 (A5 + B5 + A8), 128.4 (T5), 127.2 (B8), 124.9 (T3), 123.7 T3′), 121.4 (A4), 120.8 (B4). *ES MS m/z (calc.):* 626.6 (626.1 $[M – Cl]^+$).

[Ru(tpy)(i-Hdiqa)(Cl)]Cl

$[Ru(tpy)(Cl)_3]$ (135 mg, 0.307 mmol), i-Hdiqa (100 mg, 0.369 mmol), and lithium chloride (65 mg, 1.5 mmol) were dissolved in a degassed ethanol/water mixture (3:1, 20 mL). Triethylamine (400 µL, 2.6 mmol) was added and the reaction mixture was refluxed under dinitrogen atmosphere for 4 h. The reaction mixture was filtered hot over *Celite* and the cake was washed with ethanol until the filtrate was colorless. After evaporation of the solvents, the crude product was purified by column chromatography on silica with dichloromethane/methanol (9:1) as eluent (R_f = 0.42), to yield a dark reddish brown solid. Yield: 83% (173 mg, 0.256 mmol).

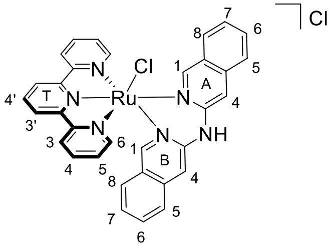

^{1}H NMR (300 MHz, methanol-d_4, 298 K) δ 10.35 (s, 1H, A1), 8.61 (d, *J* = 8.1 Hz, 2H, T3′), 8.61 – 8.56 (m, 2H, T6), 8.55 (dd, *J* = 8.0, 1.2 Hz, 2H, T3), 8.14 (dd, *J* = 8.4, 1.1 Hz, 1H, A8), 8.10 (t, *J* = 8.1 Hz, 1H, T4′), 8.03 (dd, *J* = 8.3, 1.0 Hz, 1H, A5), 8.01 (ddd, *J* = 8.0, 7.8, 1.5 Hz, 2H, T4), 7.85 (ddd, *J* = 8.3, 6.9, 1.1 Hz, 1H, A6), 7.84 (s, 1H, A4), 7.64 (ddd, *J* = 8.4, 6.9, 1.0 Hz, 1H, A7), 7.59 (dd, *J* = 8.1, 1.1 Hz, 1H, B5), 7.56 – 7.51 (m, 2H, T5), 7.50 (s, 1H, B1), 7.51 – 7.47 (m, 1H, B6), 7.31 (dd, *J* = 8.3, 1.0 Hz, 1H, B8), 7.23 (s, 1H, B4), 7.23 (ddd, *J* = 8.3, 6.6, 1.1 Hz, 1H, B7). *^{13}C NMR (75 MHz, methanol-d_4, 298 K)* δ 160.8 + 160.8 (C_q T2 + T2′), 160.1 (A1), 154.6 (T6), 154.4 (B1), 151.3 (C_q A3 or B3), 139.6 (C_q A4a or B4a), 138.4 (T4), 135.2 (T4′), 133.6 (A6), 133.4 (B6), 128.8 (A8), 128.3 (T5), 127.9 + 126.9 (C_q A8a + B8a), 127.7 (A7), 127.4 (B7), 127.1 (B8), 126.8 (A5), 126.2 (B5), 124.9 (T3), 123.7 (T3′), 108.0 (A4), 107.3 (B4), two quaternary carbons are missing: C_q A3 or B3, C_q A4a or B4a. *ES MS m/z (calc.):* 641.6 (641.1 $[M – Cl]^+$).

$[Ru(tpy)(i\text{-}biq)(Hmte)](PF_6)_2$, $[2](PF_6)_2$

[Ru(tpy)(i-biq)(Cl)]Cl (21 mg, 0.032 mmol) and $AgPF_6$ (17 mg, 0.067 mmol) were dissolved in a degassed acetone/water mixture (3:5, 16 mL). 2-(Methylthio)ethanol (138 µL, 1.53 mmol) was added in excess to the reaction mixture. The reaction was stirred and heated to reflux under dinitrogen atmosphere for 4 h, filtered hot over *Celite*, and the cake was washed with acetone until the filtrate was colorless. The solvents were removed by rotary evaporation. The product was dissolved in a minimum amount of acetone and reprecipitated by addition to an excess of diethyl ether. Filtration yielded the final product, which was dried in air and then under vacuum as a bright orange powder. Yield: 48% (15 mg, 0.015 mmol).

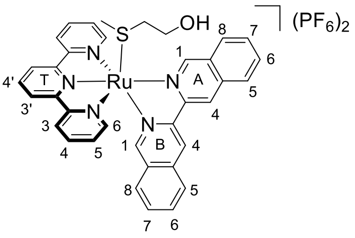

^{1}H NMR (300 MHz, acetone-d$_6$, 298 K) δ (ppm) 10.64 (s, 1H, A1), 9.54 (s, 1H, A4), 9.32 (s, 1H, B4), 8.99 (d, *J* = 8.1 Hz, 2H, T3′), 8.79 (dd, *J* = 8.0, 1.3 Hz, 2H, T3), 8.57 (t, *J* = 8.1 Hz, 1H, T4′), 8.54 (d, *J* = 8.3 Hz, 2H, A8), 8.42 (d, *J* = 8.2 Hz, 1H, A5), 8.27 – 8.22 (m, 3H, B1 + T6), 8.17 – 8.09 (m, 4H, T4 + B5 + A6), 8.04 (ddd, *J* = 8.2, 7.0, 1.2 Hz, 1H, A7), 7.84 (ddd, *J* = 8.2, 6.7, 1.4 Hz, 1H, B6), 7.71 (d, *J* = 8.0 Hz, 1H, B8), 7.63 (ddd, *J* = 8.2, 6.7, 1.0 Hz, 1H, B7), 7.47 (ddd, *J* = 7.7, 5.5, 1.3 Hz, 2H, T5), 4.77 (t, *J* = 4.7 Hz, 1H, O<u>H</u>), 3.59 (dt, *J* = 5.0, 4.7 Hz, 2H, S-CH$_2$-C<u>H$_2$</u>), 2.10 (t, *J* = 5.0 Hz, 2H, S-C<u>H$_2$</u>), 1.54 (s, 3H, S-C<u>H$_3$</u>). *^{13}C NMR (300 MHz, acetone-d$_6$, 298 K)* δ (ppm) 159.2 + 158.8 (C$_q$ T2 + T2′), 156.8 (A1), 154.7 (B1), 154.4 (T6), 150.8 + 150.4 (C$_q$ A3 + B3), 139.7 (T4), 137.7 (T4′), 136.7 + 136.1 (C$_q$ A4a + B4a), 134.0 (B6 + A6), 131.1 (A7), 130.8 + 129.8 (C$_q$ A8a + B8a), 130.6 (B7), 129.3 (T5), 129.1 (A8), 128.7 (A5), 128.3 (B5 + B8), 126.0 (T3), 125.3 (T3′), 122.1 (A4), 121.4 (B4), 58.8 (S-CH$_2$-<u>C</u>H$_2$), 38.4 (S-<u>C</u>H$_2$), 14.8 (S-<u>C</u>H$_3$). *High resolution ES MS m/z (calc.):* 341.5644 (341.5645, [M – 2PF$_6$]$^{2+}$). *Elem. Anal. Calc. for $C_{36}H_{31}F_{12}N_5OP_2RuS$:* C, 44.45; H, 3.21; N, 7.20. *Found:* C, 43.75; H, 3.30; N, 7.12.

[Ru(tpy)(i-Hdiqa)(Hmte)](PF$_6$)$_2$, [3](PF$_6$)$_2$

[Ru(tpy)(i-Hdiqa)(Cl)]Cl (150 mg, 0.222 mmol) and AgPF$_6$ (123 mg, 0.488 mmol) were dissolved in a degassed acetone/water mixture (3:5, 30 mL). 2-(Methylthio)ethanol (1 mL, 0.01 mol) was added in excess to the reaction mixture. The reaction was stirred and heated to reflux under dinitrogen atmosphere for 3 h, filtered hot over *Celite*, and the cake was washed with acetone until the filtrate was colorless. The solvents were removed by rotary evaporation. The product was dissolved in a minimum amount of acetone and precipitated by addition to an excess of diethyl ether. Filtration yielded the final product as an orange powder, which was dried in air and then under vacuum. Yield: 60% (132 mg, 0.134 mmol).

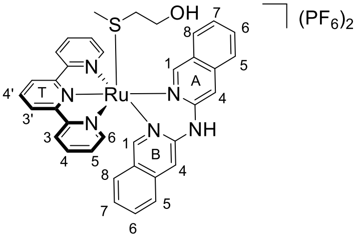

^{1}H NMR (300 MHz, acetone-d$_6$, 298 K) δ (ppm) 10.15 (s, 1H, A1), 10.01 (s, 1H, N<u>H</u>), 8.95 (dd, *J* = 5.6, 0.8 Hz, 2H, T6), 8.89 (d, *J* = 8.2 Hz, 2H, T3′), 8.77 (dd, *J* = 8.1, 0.7 Hz, 2H, T3), 8.46 (t, *J* = 8.2 Hz, 1H, T4′), 8.35 (d, *J* = 8.3 Hz, 1H, A8), 8.27 (ddd, *J* = 8.1, 7.8, 0.8 Hz, 2H, T4), 8.15 (s, 1H, A4), 8.10 (d, *J* = 8.4 Hz 1H, A5), 7.93 (ddd, *J* = 8.3, 6.8, 1.2 Hz, 1H, A6), 7.79 (s, 1H, B1), 7.78 – 7.68 (m, 4H, T5 + A7 + B5), 7.65 – 7.58 (m, 2H, B4 + B6), 7.48 (d, *J* = 8.3 Hz, 1H, B8), 7.30 (ddd, *J* = 8.3, 6.7, 0.9 Hz, 1H, B7), 4.13 (t, *J* = 5.2 Hz, 1H, -O<u>H</u>), 3.49 (dt, *J* = 5.6, 5.2 Hz, 2H, S-CH$_2$-C<u>H$_2$</u>), 1.91 (t, *J* = 5.6 Hz, 2H, S-C<u>H$_2$</u>), 1.37 (s, 3H, S-C<u>H$_3$</u>). *^{13}C NMR (300 MHz, acetone-d$_6$, 298 K)* δ (ppm) 159.4 + 159.3 (C$_q$ T2 + T2′), 159.0 (A1), 155.7 (T6), 152.7 (B1), 151.4 + 151.0 (C$_q$ A3 + B3), 139.8 (T4), 139.4 + 138.6 (C$_q$ A4a + B4a), 137.4 (T4′), 133.9 (A6), 133.9 (B6), 129.1 (T5), 128.9 (A8), 128.2 + 126.7 (C$_q$ A8a + B8a), 128.1 (B8), 127.8 (A7), 127.3 (B7), 126.7 (A5), 126.1 (B5), 126.0 (T3), 125.3 (T3′), 110.2 (A4), 108.7 (B4), 59.0 (S-CH$_2$-<u>C</u>H$_2$), 38.0 (S-<u>C</u>H$_2$), 15.1 (S-<u>C</u>H$_3$). *High resolution ES MS m/z (calc.):* 349.0698 (349.0699, [M – 2PF$_6$]$^{2+}$). *Elem. Anal. Calc. for $C_{36}H_{32}F_{12}N_6OP_2RuS$ + 3 H_2O:* C, 41.51; H, 3.68; N, 8.07. *Found:* C, 41.77; H, 3.45; N, 8.11.

2. Single Crystal X-Ray crystallography

Single crystals of [**2**]$(PF_6)_2$ were obtained by recrystallization through liquid-vapor diffusion using acetonitrile as solvent and diisopropyl ether as counter-solvent. In short, 1 mg of [**2**]$(PF_6)_2$ was dissolved in acetonitrile (1 mL) and placed in a small vial. This vial was placed in a larger vial containing diisopropyl ether (2.8 mL). The large vial was closed and vapor diffusion within a few days afforded X-ray quality crystals.

All reflection intensities were measured at 110(2) K using a SuperNova diffractometer (equipped with Atlas detector) with Cu $K\alpha$ radiation (λ = 1.54178 Å) under the program CrysAlisPro (Version CrysAlisPro 1.171.39.29c, Rigaku OD, 2017). The same program was used to refine the cell dimensions and for data reduction. The structure was solved with the program SHELXS-2014/7 (Sheldrick, 2015) and was refined on F^2 with SHELXL-2014/7 (Sheldrick, 2015). Analytical numeric absorption correction using a multifaceted crystal model was applied using CrysAlisPro. The temperature of the data collection was controlled using the system Cryojet (manufactured by Oxford Instruments). The H atoms were placed at calculated positions using the instructions AFIX 23, AFIX 43 or AFIX 137 with isotropic displacement parameters having values 1.2 or 1.5 Ueq of the attached C atoms. The H atoms attached to the disordered hydroxyl groups O1A/O1A′ and O1B/O1B′ could not be retrieved reliably from difference Fourier maps, and no AFIX 147 was used because of the disorder. The crystal refines in the space group $Pca2_1$ and is racemically twinned. The Flack parameter refines to 0.539(16).

The structure of [**2**]$(PF_6)_2$ is significantly disordered. Two of the four crystallographically independent counter ions were found to be disordered over (at least) 3 different orientations. The terpyridine ligand on one of the two ruthenium complexes is disordered over two orientations. The hydroxyl groups of the Hmte ligands for both Ru1 and Ru2 complexes are disordered over two orientations. [**2**]$(PF_6)_2$: 0.51 × 0.10 × 0.05 mm^3, Orthorhombic, $Pca2_1$, a = 22.0959 (11), b = 8.8289 (2), c = 37.3521 (9) Å, V = 7286.7 (4) Å^3, Z = 8, μ = 5.78 mm^{-1}, transmission factor range: 0.280–0.812. 23674 Reflections were measured up to a resolution of $(\sin \theta/\lambda)_{max}$ = 0.616 Å^{-1}. 11592 Reflections were unique (R_{int} = 0.037), of which 10905 were observed [$I > 2\sigma(I)$]. 1423 Parameters were refined. R1/wR2 [$I > 2\sigma(I)$]: 0.0525/ 0.1383. R1/wR2 [all refl.]: 0.0558/ 0.1407. S= 1.11. Residual electron density found between −0.87 and 1.63 e Å^{-3}.

The X-ray structure has been deposited in the Cambridge Crystallographic Data Center and can be retrieved there under the reference CCDC 2047321.

3. Log P determination by ICP-MS

Materials. 65% Nitric acid (Suprapur, Merck) was used in the sample digestion process, while diluted 1% nitric acid (v/v) was employed as a carrying solution throughout the ICP measurements. For preparation of calibration and internal standards National Institute of Standards and Technology (NIST)-traceable 1000 mg/L elemental standards were used (TraceCERT, Fluka). Approximately 18 MΩ cm^{-1} water (Milli-Q) was employed in all sample preparation and analysis steps.

Instrumentation. Calibration standards were prepared in a Secuflow fume hood (SCALA) to prevent contamination by atmospheric particulates. The standard samples and measurement samples were analyzed for trace elements using the NexION 2000 (PerkinElmer) ICP-MS instrument equipped with a concentric glass nebulizer and Peltier-cooled glass spray chamber. An SC2 DX autosampler (PerkinElmer) was connected to the ICP-MS for sample introduction. Syngistix software for ICP-MS (v.2.5, PerkinElmer) was used for all data recording and processing. Five trace elemental calibration standards for ICP-MS analysis were prepared using NIST-traceable 1000 mg/L Ru standards: 0, 1, 5, 20, and 100 μg/L. Samples were analyzed without dilution in the original delivery containers to minimize the possibility of contamination. Here, 10 μg/L Rh and In were used as internal standards. To check the calibration, samples were analyzed with a blank measurement and a repeat measurement of one of the calibration standards. For the calibration curve, the accepted correlation coefficient (Cor.Coeff) was to be found higher than 0.999.

Lipophilicity (Log P_{ow}) determination. The 1-octanol-saturated water and water-saturated 1-octanol were prepared using MilliQ water stirred with 1-octanol for 24 h at room temperature. The two layers were separated by centrifugation at 2000 rpm for 5 min.
To determine the Log P_{ow} value two different preparation procedures were applied:

Method for water-soluble compounds: [**1**]$(PF_6)_2$ was dissolved in 1-octanol-saturated water to obtain a 1 mM solution. Afterwards, the solution was centrifuged for 5 min at 2000 rpm. Five aliquots (5, 10, 50, 100 and 150 μL) of the supernatant solution were collected, transferred into 15.0 mL Corning tubes, and diluted to 1.00 mL with 1-octanol-saturated water. Then 1.00 mL of water-saturated 1-octanol was added to each sample.

Method for water-insoluble compounds: [**2**]$(PF_6)_2$ and [**3**]$(PF_6)_2$ were dissolved in water-saturated 1-octanol to obtain a 1 mM solution and heated at 100 °C for 10 min. Afterwards, the solution was centrifuged for 5 min at 2000 rpm. Five aliquots (5, 10, 50, 100 and 150 μL) of the supernatant were collected, transferred into five 15.0 mL Corning tubes, and diluted to 1.0 mL with water-saturated 1-octanol. Then 1.0 mL of 1-octanol saturated water was added to each sample.

All water-octanol samples were shaken on Ika®Roller 6 at 40 rpm for 24 h at rt. Afterwards, the tubes were centrifuged at 2000 rpm for 5 min obtaining a clear separation between the two layers in each tube. 500 μL aliquots from the aqueous phase were transferred to a new Corning 15.0 mL tube, digested using 500 μL of 65% HNO_3, mixed, and then diluted with 9.0 mL of Milli-Q water to make total volume to 10.0 mL. 10 μL of each starting stock solution of complexes [**1**]$(PF_6)_2$, [**2**]$(PF_6)_2$ or [**3**]$(PF_6)_2$ was digested by adding 490 μL of 65% HNO_3 and shaking for 1 h. Subsequently, each stock solution sample was diluted to 10.0 mL by adding Milli-Q water.

For the water-soluble complex [**1**](PF_6)$_2$ the Ru concentration in the octanol phase had to be measured; in order to do this, 500 μL of the octanol phase was transferred in a glas vial and 500 μL 65% HNO_3 were added. The glass vial has been equipped with a marble in order to prevent water evaporation, heated for 3 h at 90 °C and then diluted to 10.0 mL by adding Mili-Q water.

Then for all samples of all three complexes [**1**](PF_6)$_2$, [**2**](PF_6)$_2$ and [**3**](PF_6)$_2$ the ruthenium concentration in the water phase of each biphasic mixture, that of the stock aqueous solutions, and that of the octanol phases for [**1**](PF_6)$_2$, were examined by ICP-MS (NexION 2000, PerkinElmar). Log P_{ow} value of [**1**](PF_6)$_2$ was calculated using the following formula:

$$\text{Log } P_{\text{ow}} = \log\frac{[Ru]_{octanol}}{[Ru]_{water}}$$

Where both $[Ru]_{octanol}$ and $[Ru]_{water}$ was measured by ICP-MS, while for [**2**](PF_6)$_2$ and [**3**](PF_6)$_2$ the following formula was applied:

$$\text{Log } P_{\text{ow}} = \log\frac{[Ru]_{total}-[Ru]_{water}}{[Ru]_{water}}$$

Where $[Ru]_{total}$ is the concentration measured for the stock solution, and $[Ru]_{water}$ that of the water phase of the biphasic mixture.

Table S1. Log P_{ow} values of complexes **1**, **2** and **3**.

Complex	Log P_{ow}
[**1**](PF_6)$_2$	-3.28 ± 0.31
[**2**](PF_6)$_2$	2.10 ± 0.27
[**3**](PF_6)$_2$	0.45 ± 0.10

4. Dark stability in water and OptiMEM

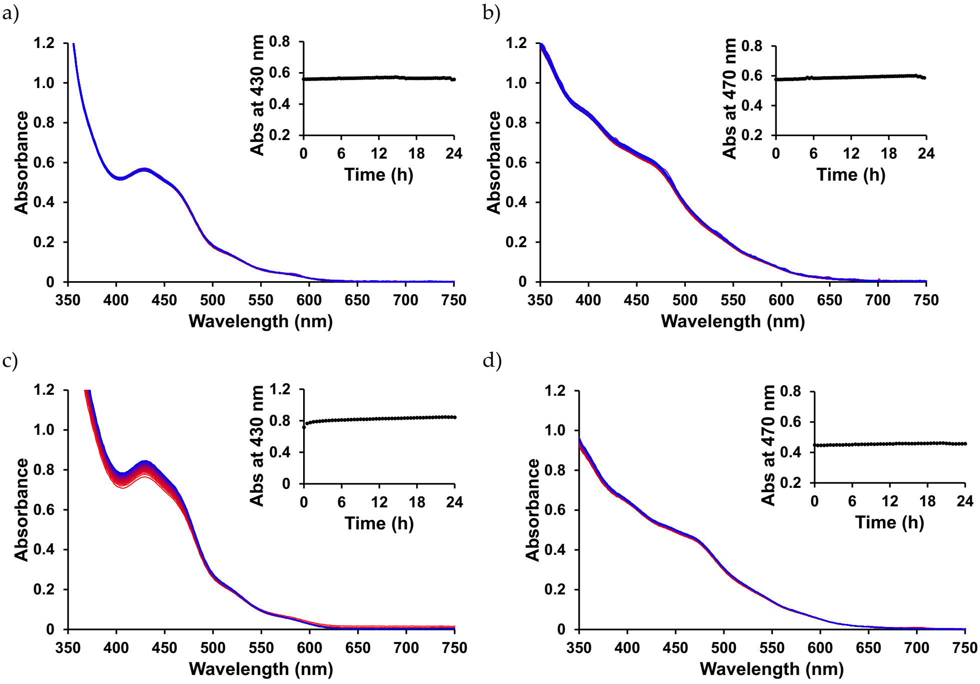

Figure S1. Evolution of the UV-vis spectra (region 350 – 750 nm) of a solution of a) [**2**](PF_6)$_2$ and b) [**3**](PF_6)$_2$ in water, and c) [**2**](PF_6)$_2$ and d) [**3**](PF_6)$_2$ in OptiMEM complete. Conditions: [Ru] = 0.097, 0.104, 133, and 0.081 mM, respectively, T = 37 °C, t = 24 h, V = 3 mL, under air atmosphere and in the dark. Inset: Time evolution of absorbance at wavelength 430nm for [**2**](PF_6)$_2$ and 470 nm for [**3**](PF_6)$_2$.

5. Molar extinction coefficient in water

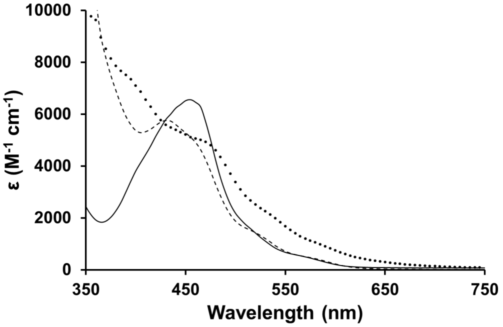

Figure S2. Molar absorbance of solutions of [**1**](PF_6)$_2$ (---), [**2**](PF_6)$_2$ (- -), and [**3**](PF_6)$_2$ (· ·) in water.

6. Singlet oxygen production and phosphorescence

Singlet oxygen quantum yield measurements were performed as described by Zhou *et al.*[4]

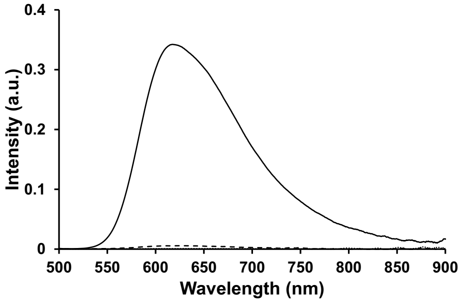

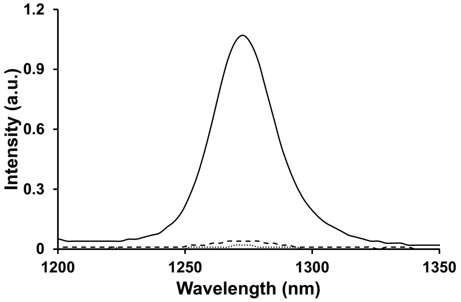

Figure S3. Visible emission spectra (left) of and near-infrared spectra of $^1O_2$ phosphorescence (λ_{em} = 1275 nm) (right) sensitized by [**2**](PF_6)$_2$ (···), [**3**](PF_6)$_2$ (- -), and [Ru(bpy)$_3$]Cl_2 (—) in aerated methanol-d$_4$ at 293 K under blue-light irradiation (450 nm, 0.4 W · cm^{-2}).

7. Irradiation experiments monitored with UV-vis and MS

Photoreactions monitored with UV-vis spectroscopy were performed on a Cary Varian spectrometer equipped with temperature control set to 310 K and a magnetic stirrer. The measurements were performed in a quartz cuvette, containing 3 mL of solution. The stirred sample was irradiated perpendicularly to the axis of the spectrometer with the beam of an LED fitted to the top of the cuvette.

For photoactivation with green light, an LED light source (λ = 517 nm, $\Delta\lambda_{1/2}$ = 23 nm, 5.2 mW) was used, an absorption spectrum was measured every 30 sec for 70 min for [**2**](PF_6)$_2$ and 47 min for [**3**](PF_6)$_2$. [Ru] = 0.074 and 0.061 mM and Φ = 5.2 · 10^{-8} mol · s^{-1} for [**2**](PF_6)$_2$ and [**3**](PF_6)$_2$. Data were analyzed using Microsoft Excel. Mass spectrometry was performed at the beginning and at the end of the irradiation to confirm the nature of the reagent and products.

8. MS of the ruthenium species after green light irradiation

a) b)

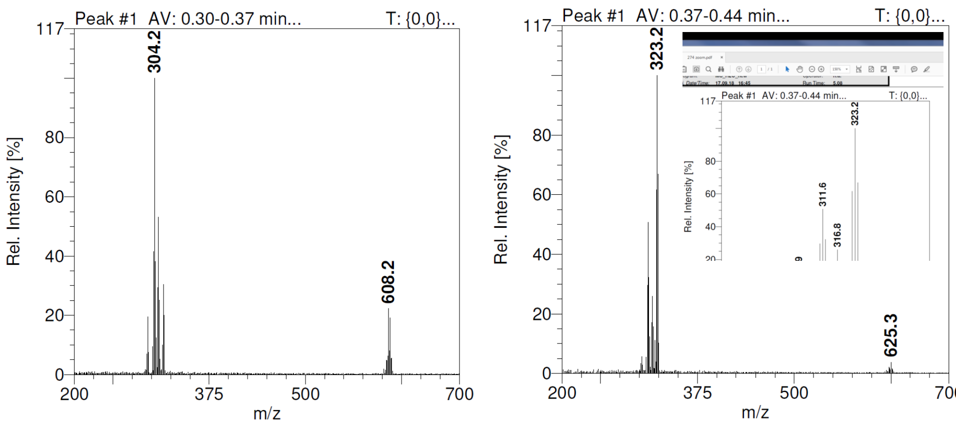

Figure S4. Mass spectrum of a solution of [**2**](PF_6)$_2$ and [**3**](PF_6)$_2$ in water after 50 min of light irradiation at 310 K with a 517 nm LED with a photon flux of Φ_{517} = 5.2 · 10^{-8} mol · s^{-1} under air atmosphere. Peaks corresponding to a) [Ru(tpy)(i-biq)(OH_2)]$^{2+}$ (calc. m/z = 304.5) and [Ru(tpy)(i-biq)(OH)]$^{+}$ (calc. m/z = 608.1); and b) [Ru(tpy)(i-Hdiqa)(OH_2)]$^{2+}$ (calc. m/z = 312.1) and [Ru(tpy)(i-Hdiqa)(OH)]$^{+}$ (calc. m/z = 623.1). [Ru(tpy)(i-Hdiqa)(MeCN)]$^{2+}$ (calc. m/z = 323.6).

9. Photosubstitution quantum yield simulated by Glotaran

Photosubstitution quantum yield calculations were performed using the Glotaran Software package. The conditions are summarized in Table S1.

Table S2. Conditions of the photoreactions used for Glotaran calculations.

	**[2](PF_6)$_2$**	**[3](PF_6)$_2$**
irradiation wavelength (λ in nm)	517	517
volume (V in L)	0.003	0.003
path length (l in m)	0.01	0.01
concentration (c in M)	$7.41 \cdot 10^{-5}$	$6.11 \cdot 10^{-5}$
photon flux (Φ in mol $\cdot$ s^{-1})	$5.2 \cdot 10^{-8}$	$5.2 \cdot 10^{-8}$
epsilon Ru-L (ε in $M^{-1} \cdot cm^{-1}$) at 517 nm	1435	2651
epsilon Ru-OH_2 (ε in $M^{-1} \cdot cm^{-1}$) at 517 nm	3305	5025

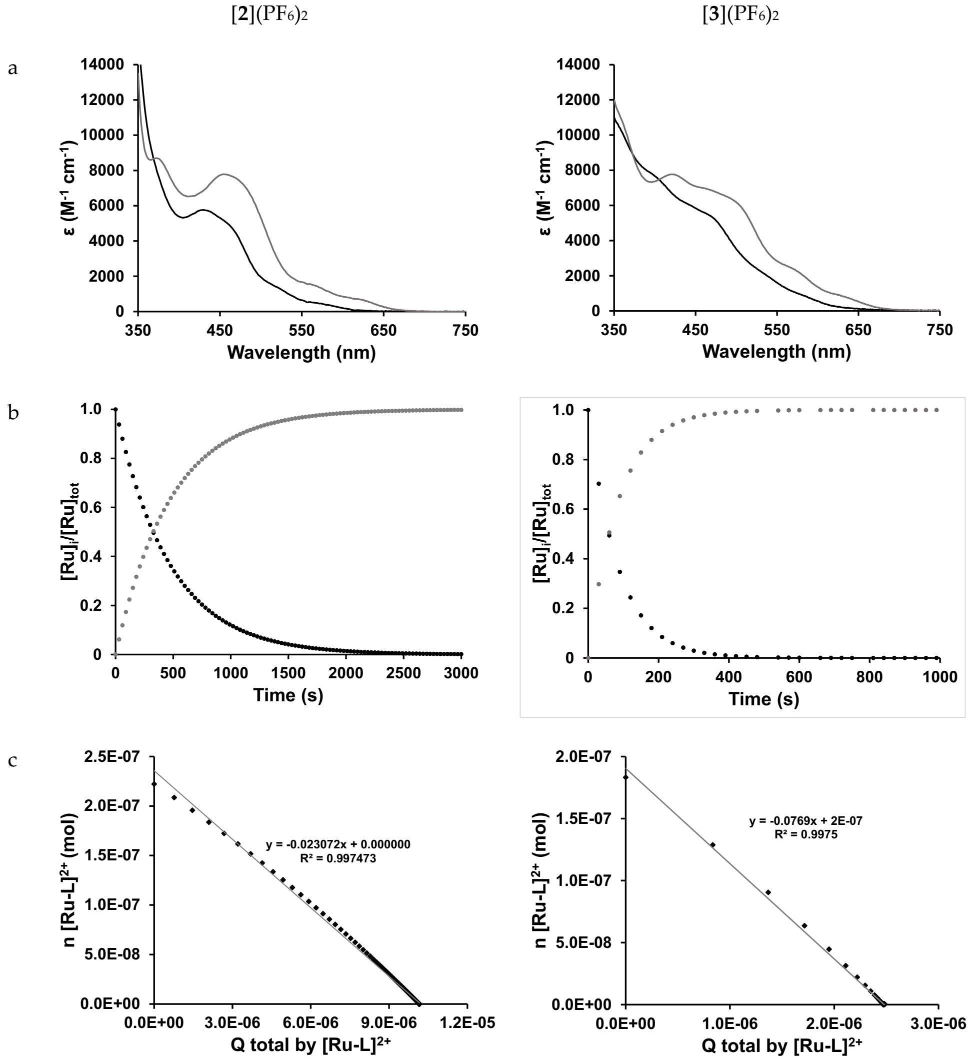

Figure S5. Kinetic data for the photosubstitution of Hmte according to the time evolution of the absorbance spectra of solutions of [**2**]$(PF_6)_2$ and [**3**]$(PF_6)_2$ in H_2O irradiated with green light under air atmosphere. a) Globally fitted absorption spectra of the starting material [**2**]$(PF_6)_2$ and [**3**]$(PF_6)_2$ (black) and their aqua products $[Ru(tpy)(NN)(H_2O)]^{2+}$ ([**5**]$^{2+}$ and [**6**]$^{2+}$, grey). b) Modelled evolution of the relative concentration of [**2**]$^{2+}$ and [**3**]$^{2+}$ *vs.* irradiation time according to global fitting using Glotaran. c) Plot of the amount of [**2**]$^{2+}$ and [**3**]$^{2+}$ (mol) *vs.* total amount of photons absorbed by [**2**]$^{2+}$ and [**3**]$^{2+}$ since t = 0 (mol). The slope of the obtained line is the opposite of the quantum yield of the formation for the aqua complex. Conditions: 0.074 and 0.061 mM solution of [**2**]$(PF_6)_2$ and [**3**]$(PF_6)_2$ in MilliQ H_2O irradiated at 298 K under air atmosphere using a 517 nm LED.

10. Cell culture and EC_{50} (photo)cytotoxicity studies

Materials

Human cancer cell line A549 (human lung carcinoma) and A431 (human epidormoid carcinoma) were distributed by the European Collection of Cell Cultures (ECACC) and purchased from Sigma Aldrich. Dulbecco's Modified Eagle Medium (DMEM, without phenol red, without glutamine), Glutamine-S (GM; 200 mM), trichloroacetic acid (TCA), glacial acetic acid, sulforhodamine B (SRB), and tris(hydroxylmethyl)aminomethane (Trisbase) were purchased from Sigma Aldrich. Fetal calf serum (FCS) was purchased from Hyclone. Penicillin and streptomycin were purchased from Duchefa and were diluted to a 100 mg/mL penicillin/streptomycin solution (P/S). Trypsin and OptiMEM (without phenol red) were purchased from Gibco Life Technologies. Trypan blue (0.4% in 0.81% sodium chloride and 0.06% potassium phosphate dibasic solution) was purchased from BioRad. Plastic disposable flasks and 96-well plates for cytotoxicity assays were purchased from Sarstedt. Cells were counted by using a BioRad TC10 automated cell counter with Biorad cell-counting slides. Cells were inspected with an Olympus IX81 microscope. UV-vis measurements for analysis of 96-well plates were performed with a M1000 Tecan Reader.

Cell Culturing

Cells were cultured in Dulbecco's Modified Eagle Medium containing phenol red, supplemented with 9.0% v/v FCS, 0.2% v/v P/S and 0.9% v/v GM (called DMEM complete) and incubated at 37 ºC at 7.0% CO_2 in 75 cm^2 T-flasks. Fresh cells were passaged at least twice after being thawed and splitted once a week at 80-90% confluency. Cells were cultured for a maxium of 8 weeks for all biological experiment.

(Photo)cytotoxicity assays

For each photocytotoxicity experiment, a parallel control plate was prepared and treated identically, but without irradiation. A549 and A431 cells were seeded at t = 0 in 96-well plates at a density of 5000 and 8000 cells/well (100 μL), respectively in OptiMEM supplemented with 2.4% v/v FCS, 0.2% v/v P/S, and 1.0% v/v GM (called OptiMEM complete) and incubated for 24 h at 37 ºC and 7.0% CO_2. Only the inner 60 wells were used for seeding, the outer wells were kept cell free to prevent border effects during irradiation. At t = 24 h, aliquots (100 μL) of six different concentrations of freshly prepared stock solutions of the compounds in OptiMEM complete were added to the wells in triplicate (see plate design in Figure I.3) and incubated for 24 h. Sterilized dimethylsulfoxide (DMSO) was used to dissolve the compounds in such amounts that the maximum v/v% of DMSO per well did not exceed 0.5%. At t = 48 h, the plates were irradiated with the cell-irradiation setup (520 nm, 30 min, 38 J/cm^2) and the control plate was kept in the dark. After irradiation, all the plates were incubated in the dark until a total time of t = 96 h after seeding. The cells were fixated by adding cold TCA (10% w/v; 100 μL) in each well and the plates were stored at 4 ºC for at least 4 h as part of the SRB assay that was adapted from Vichai *et al.*[5] In short, after fixation, the TCA medium mixture was removed from the wells, rinsed with demineralized water three times. Then, each well was stained with 100 μL SRB (0.6% w/v in 1% v/v acetic acid) for 30 min, the SRB was removed by washing with acetic acid (1% v/v), and air dried. The SRB dye was solubilized with Tris base (10 mM; 200 μL) overnight, and the absorbance in each well was read at λ = 510 nm.

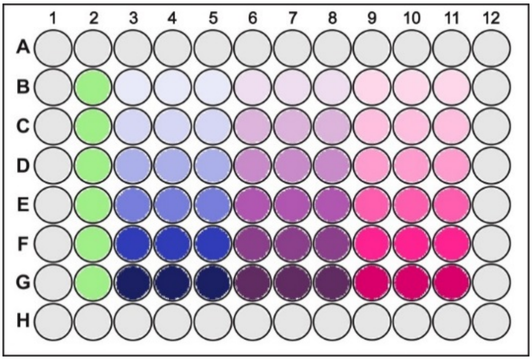

Figure S6. Design of a 96-well plate used in the (photo)cytotoxicity assays. Grey: Outer wells are not used for seeding to prevent border effects; green: non-treated cells ($n_t = 6$); blue: cells treated with compound A; purple: cells treated with compound B; pink: cells treated with compound C. Each compound was added in six different concentrations (one per row) per triplicate ($n_t = 3$).

The SRB absorbance data per compound per concentration was averaged over three identical wells (technical replicates, $n_t = 3$) in Excel and was exported to GraphPad Prism. Relative cell populations were calculated by dividing the average absorbance of the treated wells by the average absorbance of the untreated wells. It was checked that the cell viability of the untreated cells of the samples irradiated were similar (maximum difference of 10%) to the non-irradiated samples to make sure no harm was done by light alone. The resulting dose-response curve for each compound under dark and irradiated conditions was fitted to a non-linear regression function with fixed y maximum (100%) and minimum (0%) (relative cell viability) and a variable Hill slope. The data of three independent biological replications was used to obtain the effective concentrations (EC_{50} in μM). Photo indices (PI) were calculated, for each compound, by dividing the EC_{50} value obtained in the dark by the EC_{50} value determined under light irradiation.

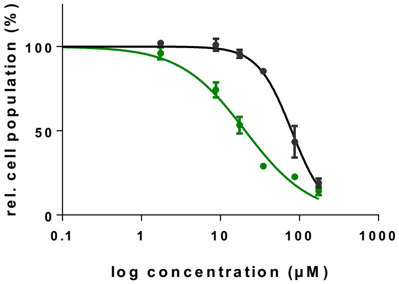

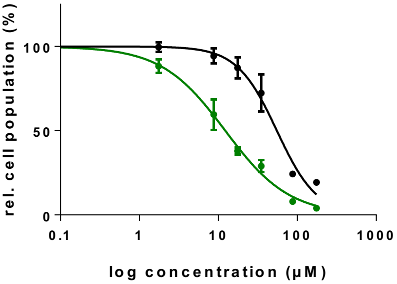

Figure S7. Dose response curves for A549 (left) and A431 (right) cells under normoxia treated with [**2**]$(PF_6)_2$ and irradiated with green light (520 nm, 38 J · cm^{-2}) 24 h after treatment (green line) or left in the dark (black line).

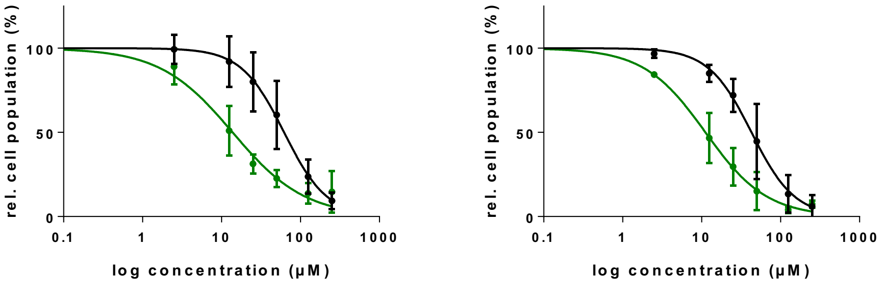

Figure S8. Dose response curves for A549 (left) and A431 (right) cells under normoxia treated with [**3**]$(PF_6)_2$ and irradiated with green light (520 nm, 38 J · cm^{-2}) 24 h after treatment (green line) or left in the dark (black line).

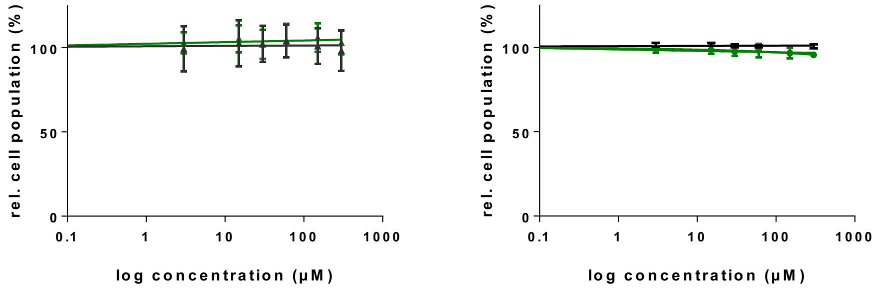

Figure S9. Dose response curves for A549 (left) and A431 (right) cells under normoxia treated with Hmte and irradiated with green light (520 nm, 38 J · cm^{-2}) 24 h after treatment (green line) or left in the dark (black line).

11. Green light irradiation in the cell irradiation setup

Cell-irradiation setup

The cell-irradiation system consisted of a Ditabis thermostat (980923001) fitted with two flat-bottomed micro-plate thermoblocks (800010600) and a 96-LED array fitted to a standard 96-well plate. The 520 nm LED (OVL-3324), fans (40 mm, 24 VDC, 9714839), and power supply (EA-PS 2042-06B) were obtained from Farnell. See Hopkins *et al.* for a full description.[6]

Determination of irradiation times

To determine which light dose is necessary to fully activate the complexes during the cytotoxicity assay, the photochemical reactivity of the ruthenium-based complexes was tested. Therefore, the inner 60 wells of a 96-well plate were filled with OptiMEM complete (100 μL, seeding without cells), and aliquots of the complexes (at their highest concentration used in the cytotoxicity assay, 250 μM) were added to the first column. The plate was irradiated for a certain amount of time, hereafter a new column was filled, and the plate was irradiated again. This process was repeated several times (irradiation times: 15, 15, 5, 5, and 5 min), and was finished with the last column filled but not irradiated. In this way, the columns were irradiated cumulative for a total time of 0, 5, 10, 15, 30, and 45 min, respectively. The absorbance of each well was measured (between 350 and 700 nm) by a M1000Tecan Reader, and corrected for the absorbance of OptiMEM complete. The data was analyzed using Excel and the absorbance as function of time was plotted to check the time necessary for full activation.

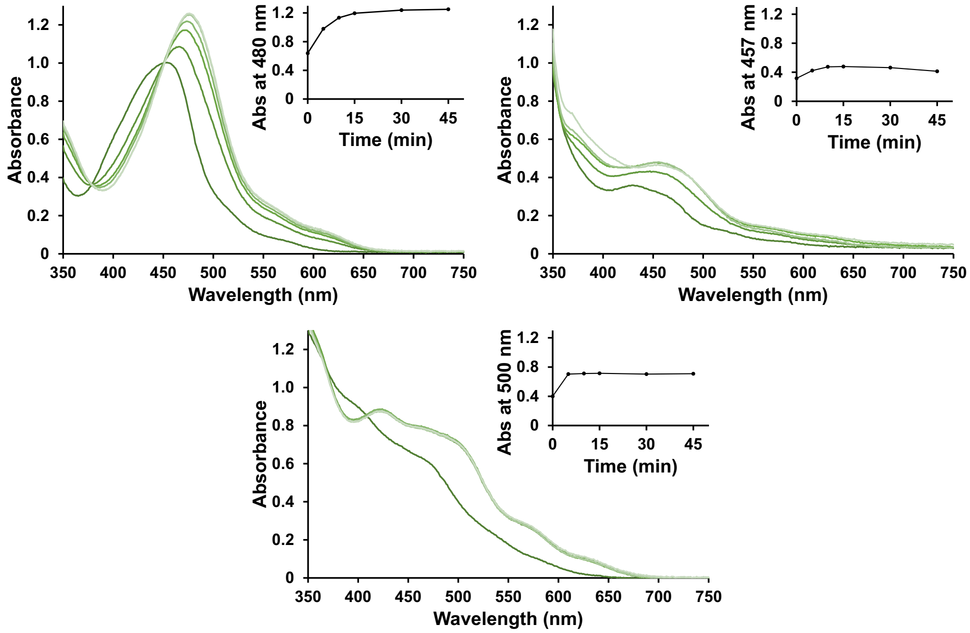

Figure S10. Evolution of the UV-vis spectra (region 350 – 750 nm) of solutions of [**1**]$(PF_6)_2$, [**2**]$(PF_6)_2$, and [**3**]$(PF_6)_2$ in demineralized water upon green light irradiation in a 96 well plate, *i.e.* under the conditions

of the cytotoxicity experiment. Conditions: [Ru] = 250 μM, T = 37 °C, t = 0, 5, 10, 15, 30, and 45 min, light source: λ = 520 ± 20 nm, 20.9 ± 1.6 mW · cm^{-2}, V = 200 μL, under air atmosphere. Inset: Time dependent absorbance at wavelength 480 nm for [**1**]$(PF_6)_2$, 457 nm for [**2**]$(PF_6)_2$, and 500 nm for [**3**]$(PF_6)_2$.

12. Cellular uptake

Cell uptake studies for the ruthenium-based complexes were conducted on A549 cancer cells at 37 °C and 21% O_2. Per compound, 1.6 10^6 cells were seeded in 10 mL OptiMEM complete in a 75 cm^2 flask at t = 0 h. At t = 24 h, the media was aspirated and the cells were treated with solutions of the complexes in 12 mL OptiMEM complete at a concentration of 30 μM. Treatment at the same concentration for all complexes allows for comparison of the amount of ruthenium taken up by the cells. 30 μM correlates to the lowest EC_{50} value of all complexes in the dark (EC_{50} value of [Ru(HCC-tpy)(i-Hdiqa)(Hmte)]$(PF_6)_2$). At t = 48 h, the medium was aspirated and the cells were washed twice with PBS (5 mL). The cells were trypsinized (2 mL, 5 min), suspended in OptiMEM complete (8 mL), and centrifuged (4 min, 1200 rpm). The supernatant was removed, the cells were resuspended in PBS (1 mL), and the cell count determined. The cells were centrifuged for a second time (4 min, 1200 rpm), the supernatant was aspirated, and the cell pellet stored at -80 °C.

For metal and protein quantification, the pellets were resuspended in demineralized water (200 μL) and lysed for 30 min by ultrasonication. The protein content of cell lysates was determined by the Bradford method. For the ruthenium measurements a contrAA 700 high-resolution continuum-source atomic absorption spectrometer (Analytik Jena AG) was used. All reagents were purchased from Sigma Aldrich. Stock solutions of the respective complexes in graded concentrations (solvent: DMSO) were used as standards and calibration was done in a matrix-matched manner. Accordingly, all samples and standards were adjusted to the same cellular protein concentration (1.0 mg cell protein per mL) by dilution (final DMSO concentration: 0.5 %). Triton X-100 (1%, 10 μL) as well as nitric acid (13%, 10 μL), were added to each standard sample (120 μL). Samples were injected (50 μL) into coated standard graphite tubes (Analytik Jena AG) and thermally processed as previously described by Skiba *et al.*[7] Drying steps were adjusted and the atomization temperature set to 2400 °C. Ruthenium was quantified at a wavelength of 349.90 nm. The mean integrated absorbance of double injections was used throughout the measurements. The data of three independent biological replications was used to obtain the uptake values, calculated as nmol metal (ruthenium) per mg cell protein.

13. DFT and TDDFT calculations

DFT was used to perform electronic structure calculations. The structure of [**2**]$^{2+}$ and [**3**]$^{2+}$ was optimized using ADF from SCM,[8] using the PBE0 hybrid functional, a triple zeta basis set (TZP) for all atoms, and COSMO to simulate solvent effects in water.

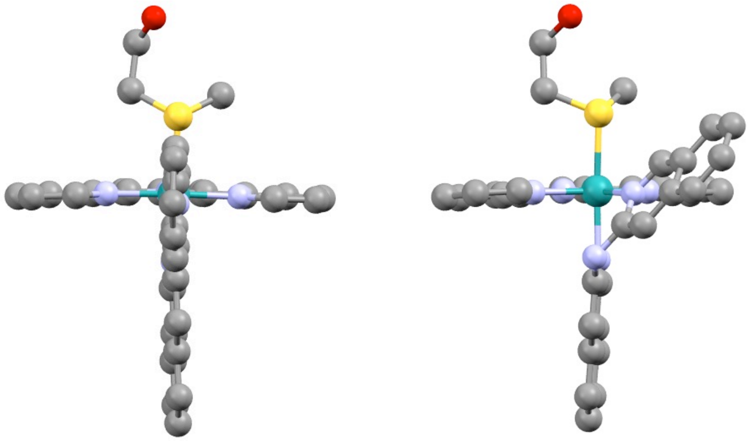

Figure S11. Structure of [2]$^{2+}$ (left) and [3]$^{2+}$ (right) optimized by DFT in water (PBE0/TZP/COSMO).

Table S3. Lowest-energy (λ>400 nm) and most intense (f>0.01) TDDFT singlet-singlet transitions calculated for [**1**]$^{2+}$, [**2**]$^{2+}$, and [**3**]$^{2+}$.

**Complex**	**Energy (nm)**	**Energy (eV)**	**Oscillator strength (f)**	**Orbital transition contribution**
[**1**]$^{2+}$	451	2.75	0.0296	HOMO-2→LUMO (52%), HOMO-1→LUMO (32%)
	430	2.88	0.1033	HOMO-2→LUMO (41%), HOMO-1→LUMO (30%)
	411	3.01	0.0172	HOMO→LUMO+2 (95%)
	405	3.06	0.0257	HOMO→LUMO+1 (62%), HOMO-1→LUMO+2 (31%)
	403	3.08	0.0290	HOMO-1→LUMO+2 (36%), HOMO-2→LUMO+2 (34%)
	401	3.09	0.0478	HOMO-1→LUMO+1 (96%)
[**2**]$^{2+}$	461	2.69	0.0322	HOMO-1→LUMO (82%)
	431	2.88	0.0717	HOMO-2→LUMO (73%)
	405	3.06	0.0798	HOMO-1→LUMO+1 (48%), HOMO-2→LUMO+1 (40%)
[**3**]$^{2+}$	476	2.60	0.0379	HOMO-1→LUMO (54%), HOMO→LUMO (27%)
	455	2.73	0.0264	HOMO-2→LUMO (85%)
	441	2.81	0.0738	HOMO-3→LUMO (48%), HOMO→LUMO (36%)
	421	2.95	0.0598	HOMO→LUMO+1 (52%), HOMO-1→LUMO+1 (43%)

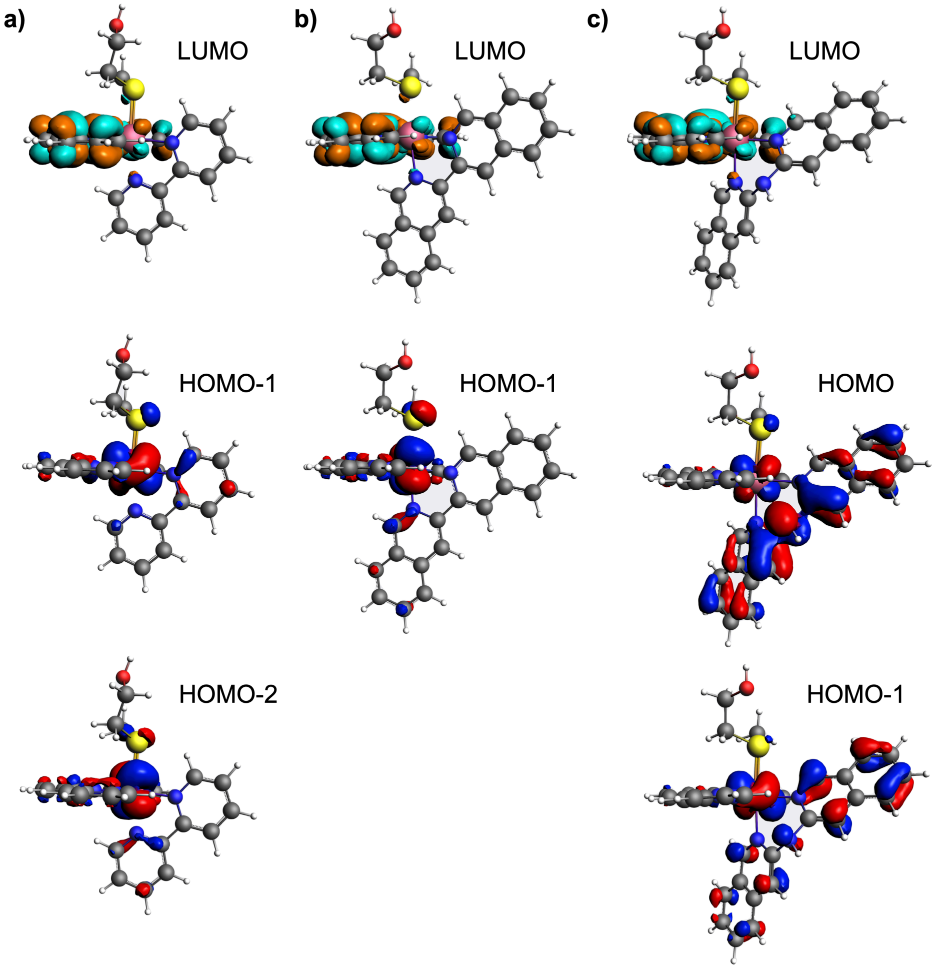

Figure S12. Frontier orbitals involved in the TDDFT-calculated lowest-energy transitions of [**1**]$^{2+}$, [**2**]$^{2+}$, and [**3**]$^{2+}$. See Table S3 for the contribution of the different orbitals to the different transitions.

14. References

1 A. Bahreman, B. Limburg, M. A. Siegler, E. Bouwman and S. Bonnet (2013) Inorg Chem 52:9456-9469

2 T. Funayama, M. Kato, H. Kosugi, M. Yagi, J. Higuchi and S. Yamauchi (2000) Bulletin of the Chemical Society of Japan 73:1541-1550

3 R. Marion, F. Sguerra, F. Di Meo, E. Sauvageot, J.-F. Lohier, R. Daniellou, J.-L. Renaud, M. Linares, M. Hamel and S. Gaillard (2014) Inorganic Chemistry 53:9181-9191

4 X.-Q. Zhou, A. Busemann, M. S. Meijer, M. A. Siegler and S. Bonnet (2019) Chemical Communications 55:4695-4698

5 V. Vichai and K. Kirtikara (2006) Nature Protocols 1:1112-1116

6 S. Hopkins, B. Siewert, S. Askes, P. Veldhuizen, R. Zwier, M. Heger and S. Bonnet (2016) Photochemical & Photobiological Sciences 15:644-653

7 J. Skiba, C. Schmidt, P. Lippmann, P. Ensslen, H.-A. Wagenknecht, R. Czerwieniec, F. Brandl, I. Ott, T. Bernaś, B. Krawczyk, D. Szczukocki and K. Kowalski (2017) European Journal of Inorganic Chemistry 2017:297-305

8 G. te Velde, F. M. Bickelhaupt, E. J. Baerends, C. Fonseca Guerra, S. J. A. van Gisbergen, J. G. Snijders and T. Ziegler (2001) Journal of Computational Chemistry 22:931-967
